# Ultrahigh‐Uptake Capacity‐Enabled Gas Separation and Fruit Preservation by a New Single‐Walled Nickel–Organic Framework

**DOI:** 10.1002/advs.202003141

**Published:** 2021-05-01

**Authors:** Yong‐Peng Li, Yong‐Ni Zhao, Shu‐Ni Li, Da‐Qiang Yuan, Yu‐Cheng Jiang, Xianhui Bu, Man‐Cheng Hu, Quan‐Guo Zhai

**Affiliations:** ^1^ Key Laboratory of Macromolecular Science of Shaanxi Province Key Laboratory of Applied Surface and Colloid Chemistry Ministry of Education School of Chemistry and Chemical Engineering Shaanxi Normal University Xi'an Shaanxi 710062 China; ^2^ School of Chemistry and Chemical Engineering Institute of Applied Catalysis Yantai University Yantai Shandong 264005 China; ^3^ Fujian Institute of Research on the Structure of Matter Chinese Academy of Sciences Fuzhou Fujian 350002 China; ^4^ Department of Chemistry and Biochemistry California State University Long Beach CA 90840 USA

**Keywords:** food preservation, high gas capture capacity, MOF synthesis, MOF structure, nickel metal–organic frameworks

## Abstract

High gas‐uptake capacity is desirable for many reasons such as gas storage and sequestration. Moreover, ultrahigh capacity can enable a practical separation process by mitigating the selectivity factor that sometimes compromises separation efficiency. Herein, a single‐walled nickel–organic framework with an exceptionally high gas capture capability is reported. For example, C_2_H_4_ and C_2_H_6_ uptake capacities are at record‐setting levels of 224 and 289 cm^3^ g^−1^ at 273 K and 1 bar (169 and 110 cm^3^ g^−1^ at 298 K and 1 bar), respectively. Such ultrahigh capacities for both gases give rise to an excellent separation performance, as shown for C_2_H_6_/C_2_H_4_ with breakthrough times of 100, 60 and 30 min at 273, 283 and 298 K and under 1 atm. This new material is also shown to readily remove ethylene released from fruits, and once again, its ultrahigh capacity plays a key role in the extraordinary length of time achieved in the preservation of the fruit freshness.

## Graphical abstract

Long live bananas! A stable nickel metal–organic framework with a voracious and unsurpassed appetite for ethane can also perform direct air capture of ethylene to keep bananas going on and on.

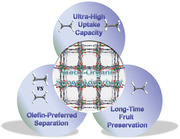

Ethylene (C_2_H_4_) is an important molecule in chemical and agricultural industries. It is the most used organic feedstock for manufacturing commodity chemicals such as polyethylene.^[^ ^1^ ^]^ Currently, storage of C_2_H_4_ is hazardous or energy‐intensive involving cryogenic temperatures and high pressure for liquefaction. When produced by steam cracking or thermal decomposition of naphtha or ethane, raw C_2_H_4_ contains C_2_H_6_ as impurity.^[^ ^2^, ^3^ ^]^ Thus, the development of energy‐efficient C_2_H_4_ purification process is among the most essential needs in chemical separations.^[^ ^4^, ^5^, ^6^, ^7^, ^8^ ^]^

C_2_H_4_ also plays a key role in regulating ripening of fruits and vegetables. Slowing down ripening by removing C_2_H_4_ can prevent food spoilage during storage and transport.^[^ ^9^, ^10^ ^]^ One approach to remove C_2_H_4_ is by oxidation with KMnO_4_.^[^ ^11^ ^]^ The development of simpler and safer physisorption‐based processes for direct air capture of C_2_H_4_ can be highly beneficial for agricultural applications.

C_2_H_4_ storage and purification using metal‐organic frameworks (MOFs) is an emerging application.^[^ ^12^, ^13^, ^14^, ^15^ ^]^ MOFs with open‐metal sites (e.g., MOF‐74 type) have been found to exhibit outstanding performance in ethylene adsorption.^[^ ^5^, ^16^, ^17^, ^18^, ^19^, ^20^, ^21^, ^22^ ^]^ However, with C_2_H_4_‐selective adsorbents, producing polymer‐grade C_2_H_4_ is still not energy efficient. In the fix‐bed C_2_H_4_/C_2_H_6_ separation with C_2_H_4_‐selective adsorbents, C_2_H_6_ comes out first and C_2_H_4_ enriched in the stationary phase is then obtained during the desorption stage. The highest C_2_H_4_ purity by such an adsorption–desorption cycle reach just about 99%. If C_2_H_6_ is preferentially adsorbed, the desired C_2_H_4_ product can be directly recovered in adsorption cycle. Compared with C_2_H_4_‐selective adsorbents, the C_2_H_6_‐selective approach can save approximately 40% of energy consumption on PSA technology.^[^ ^12^, ^23^, ^24^, ^25^, ^26^, ^27^ ^]^ So the development of new adsorbents with inverse selectivity (C_2_H_6_‐selective) is much needed.

A constant challenge in the design of adsorbent materials is the almost inevitable trade‐off between storage capacity and separation selectivity. An ideal material for separation would simultaneously have both high uptake capacity and high selectivity. But, this is especially hard to achieve for C_2_H_6_‐selective MOFs, as they tend to exhibit either poor selectivity or low uptake. Recently, Li et al. reported preferential binding of C_2_H_6_ over C_2_H_4_ by Fe_2_(O_2_)(dobdc)^[^ ^12^ ^]^ with the highest known selectivity, but it only adsorbs 74 and 58 cm^3^ g^−1^ of C_2_H_6_ and C_2_H_4_ at 293 K and 1 bar. ZIF‐7^[^ ^28^ ^]^ also selectively adsorb C_2_H_6_ over C_2_H_4_, but the adsorbed amounts of C_2_H_6_ and C_2_H_4_ were only 1.9 and 1.8 mmol g^−1^ at 100 kPa and 298 K, respectively. Another well‐known C_2_H_6_ selective MOFs is MAF‐49^[^ ^2^ ^]^ that has similar C_2_H_6_ and C_2_H_4_ uptake (1.7 mmol g^−1^) at 100 kPa and 298 K. TJT‐100^[^ ^29^ ^]^ can selectively separate C_2_H_6_ from C_2_H_6_‐C_2_H_4_ mixtures, still the C_2_H_6_ and C_2_H_4_ uptake at 100 kPa and 298 K was only about 81 and 75 cm^3^ g^−1^, respectively. Clearly, the development of a porous material simultaneously achieving high selectivity and storage performance is challenging and useful.

Here, we report a MOF super‐adsorbent (SNNU‐40) that exhibits high C_2_H_4_ and C_2_H_6_ storage capacity, useful inverse C_2_H_6_/C_2_H_4_ selectivity, and remarkable direct air capture for C_2_H_4_. This microporous single‐walled material has an unusually high uptake for C_2_H_6_ (289 cm^3^ g^–1^) and C_2_H_4_ (224 cm^3^ g^–1^) at 273 K and 1 bar that to our knowledge are the highest values among MOFs at this condition. The absence of open metal sites within SNNU‐40 results in a very low heat of adsorption (*Q*_st_) that is only about half of those values for some famous C_2_‐capture MOFs such as Cu‐TDPAT and Mg‐MOF‐74. Importantly, we show here that SNNU‐40 can preferentially adsorb ethane to achieve high‐purity ethylene with top‐level breakthrough times of 100, 60, and 30 min at 273, 283, and 303 K and 1 atm. It is also shown that SNNU‐40 exhibits excellent direct air capture ability for ethylene released from the fruit, and can prevent banana from spoilage for 30 days or more under ambient conditions.

Single‐crystal X‐ray analysis reveals that SNNU‐40 crystallizes in tetragonal space group *P*4_2_/*nmc* and the asymmetric unit consists of one Ni(II) and half of BPDC ligand (Tables S1 and S2 and Figure S1a, Supporting Information). Ni(II) displays distorted‐octahedral geometry with four O and two N atoms from BPDC ligand. Each Ni(II) is connected with four BPDC ligands through two chelating carboxylate groups and two N atoms (Figure S1b, Supporting Information) and can be regarded as an inorganic 4‐connected node. Each ligand coordinates with four metal centers through carboxylate groups and N atoms as an organic 4‐connected node (Figure 1a,b). This structure can be simplified as a 4,4‐connected network with PtS topology (**Figure** **1**d). The framework contains eight‐membered‐ring channels formed from four metal centers and four BPDC ligands. The channels alone the *c* axis has the dimensions of 7.8 × 7.8 Å^2^, while channels along the *a* and *b* axes are 4.7 × 7.4 Å^2^ and 7.8 × 9.2 Å^2^, respectively (Figure 1).

**Figure 1:**
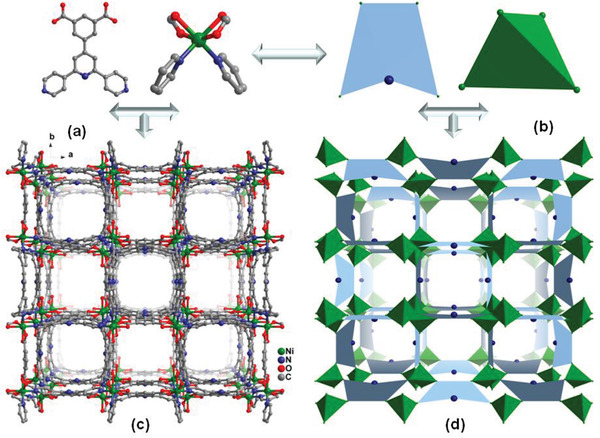
a) Structure of SNNU‐40 showing metal–ligand connection mode, b) polyhedral simplification, c) 1D channels along the *c*‐axis, and d) PtS topology.

PLATON analysis gives the free volume of 69.4%. SNNU‐40 shows remarkable chemical stability against moisture and different solvents (Figure S2, Supporting Information) according to PXRD that shows no loss of crystallinity and phase change following various treatments. Thermogravimetric analysis (TGA) shows that the framework is stable up to about 400 °C (Figures S3–S5, Supporting Information).

Desolvated SNNU‐40 was prepared by guest exchange with CH_3_CN and activation under high vacuum at 50 °C. PXRD indicates that SNNU‐40 remains crystalline (Figure S2, Supporting Information). The N_2_ sorption isotherm of desolvated SNNU‐40 at 77 K exhibits typical type‐I behavior (Figure S6, Supporting Information). The Brunauer–Emmett–Teller (BET) surface area and the Langmuir surface area from the N_2_ isotherms are 2233.8 m^2^ (STP) g^−1^ and 3484.7 m^2^ (STP) g^−1^, respectively. The pore volume of 1.22 cm^3^ (STP) g^−1^ is close to the theoretical value of 1.24 cm^3^ (STP) g^−1^ calculated from PLATON, indicating that the sample is well activated. Notably, the pore width of SNNU‐40 is 6.5 Å by Horvath–Kawazoe (H–K) method, making SNNU‐40 suitable for storage and separation of small gas molecules.

Ethane and ethylene sorption was studied at six different temperatures of 253, 263, 273, 283, 298, and 303 K up to 1 bar (**Figure** **2**a,b). No apparent hysteresis between adsorption and desorption is observed. Compared to known MOFs, SNNU‐40 exhibits exceptional performance in the uptake capacity for both C_2_H_6_ and C_2_H_4_.

**Figure 2:**
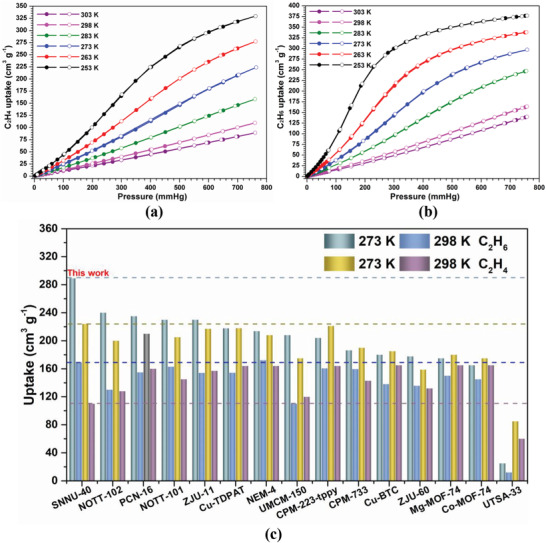
a) C_2_H_4_ and b) C_2_H_6_ adsorption isotherms of SNNU‐40 from 253 to 303 K and c) comparison between top‐level MOFs for C_2_H_4_ and C_2_H_6_ uptake.

SNNU‐40 exhibits the highest C_2_H_6_ adsorption of 289 cm^3^ g^−1^ at 273 K and 1 bar among MOFs (Figure 2c; Tables S3 and S4, Supporting Information), which far exceeds 245 cm^3^ g^−1^ for NOTT‐102^[^ ^17^, ^30^ ^]^ and 204 cm^3^ g^−1^ for CPM‐223‐tppy,^[^ ^25^ ^]^ a benchmark material for ethane uptake among ethane‐selective MOFs. As shown in Figure 2c, the uptake of SNNU‐40 for ethane at 298 K is 169 cm^3^ g^−1^, which is comparable to the highest ethane‐selective MOF of CPM‐233 (166 cm^3^ g^−1^)^[^ ^25^ ^]^ and NEM‐4 (172 cm^3^ g^−1^ at 295 K),^[^ ^31^ ^]^ and notably higher than some of the top performing MOFs, such as NOTT‐101,^[^ ^17^ ^]^ ZJU‐11,^[^ ^32^ ^]^ and PCN‐250.^[^ ^33^ ^]^

SNNU‐40 also exhibits the highest C_2_H_4_ adsorption of 224 cm^3^ g^–1^ at 273 K and 1 bar among MOFs without open metal sites in neutral frameworks (Figure 2a; Table S3, Supporting Information). The uptake value is comparable to the reported MOFs with top ethylene uptake capacity such as Cu‐TDPAT (218 cm^3^ g^−1^)^[^ ^16^ ^]^ containing both open metal sites and Lewis basic sites, PCN‐16 (210 cm^3^ g^−1^)^[^ ^17^, ^34^ ^]^ and NOTT‐101 (205 cm^3^ g^−1^)^[^ ^17^ ^]^ with open metal sites, and is even higher than those of some well‐known MOFs, like MgMOF‐74 (180 cm^3^ g^−1^).^[^ ^35^, ^36^ ^]^

At 298 K, ethylene uptake is 110 cm^3^ g^−1^ (4.9 mmol g^−1^). The weak interaction of SNNU‐40 with ethylene is advantageous for increasing the deliverable capacity (4.5 mmol g^−1^) over the pressure range of 0.1–1 bar at 298 K. SNNU‐40 ethylene deliverable is higher than Cu‐MOF‐74 (3.6 mmol g^−1^).^[^ ^36^ ^]^

A linear plot is obtained by plotting the adsorption quantity versus temperature (Figure S7, Supporting Information), the C_2_H_4_ and C_2_H_6_ uptake of SNNU‐40 decreases at a rate of 4.77 and 4.85 cm^3^ g^−1^ K^−1^ from 253 to 303 K. While the amount of C_2_H_4_ and C_2_H_6_ adsorbed decreases with increasing temperature, the preferential adsorption of the alkane with respect to the olefin does not change with temperature. Clearly, SNNU‐40 is a strong candidate for C_2_H_4_ and C_2_H_6_ capture, storage, and delivery under ambient conditions.

For practical application, we tested the repeatability of SNNU‐40 for C_2_H_4_ and C_2_H_6_ storage. Five cycles of C_2_H_4_ and C_2_H_6_ adsorption at 273 K were recorded using about 100 mg sample without reactivation between each cycle. There is no decrease in absorbed quantity of C_2_H_4_ and C_2_H_6_ after five cycles. The fully reproducible adsorption/desorption cycles indicate no oligomerization of C_2_H_4_ blocking the channels and no need for high‐temperature regeneration, which are possible downsides for MOFs with OMSs or strong adsorption sites (Figure S8, Supporting Information). Thus, SNNU‐40 is promising in refillable C_2_H_4_ and C_2_H_6_ storage.

In general, the major impurities of C_2_H_4_ are C_2_H_2_, C_2_H_6_, CO_2_, CH_4_, and N_2_;therefore, achieving efficient separation of these gases from C_2_H_4_ is required. The sorption values for pure C_2_H_2_, CO_2_, CH_4_, and N_2_ are 282.2, 118.8, 23.2, and 4.2 cm^3^ g^−1^ at 273 K and 1 bar, respectively (Figure S9, Supporting Information). The sorption values for pure C_2_H_2_, CO_2_, CH_4_, and N_2_ are 131.8, 51.7, 10.7, and 2.6 cm^3^ g^–1^ at 298 K and 1 atm, respectively (Figure S9, Supporting Information). To establish the feasibility of these gas separations, we performed calculations using the ideal adsorbed solution theory (IAST). At 298 K, the selectivity under 1 bar for the mixtures composed of equimolar binary C_2_H_2_/C_2_H_4_, C_2_H_4_/CO_2_, C_2_H_4_/CH_4_, and C_2_H_4_/N_2_ are 4.5, 1.7, 3.9, and 107.1, respectively (Figures S10–S12, Supporting Information).

The separation of C_2_H_4_ and C_2_H_6_ is a well‐known challenge. The selectivity for C_2_H_6_/C_2_H_4_ maintains a medium high value of 1.40–1.58 for a range of pressures to 100 kPa, indicating the feasibility of using SNNU‐40 for practical applications in the separation of C_2_H_4_ from C_2_H_6_. This value is comparable to ZIF‐7 (1.5),^[^ ^28^ ^]^ ZIF‐8 (1.7),^[^ ^37^ ^]^ UTSA‐33 (1.4),^[^ ^38^ ^]^ and TJT‐100 (1.2)^[^ ^29^ ^]^ at 1 bar and room temperature, but lower than Fe_2_(O_2_)(dobdc) (4.4),^[^ ^12^ ^]^ Cu(Qc)_2_ (3.4),^[^ ^39^ ^]^ and MAF‐49 (2.7)^[^ ^2^ ^]^ (**Figure** **3**c). As the temperature decreases, C_2_H_4_ production capacity increases, SNNU‐40 could produce 1.27 mmol g^–1^ C_2_H_4_ from the C_2_H_6_/C_2_H_4_ (50/50) mixture at 100 kPa and 298 K. These values are lower than that of Fe_2_O_2_(dobdc) (1.93),^[^ ^12^ ^]^ but higher than those of other prominent MOFs (Table S7, Supporting Information).

**Figure 3:**
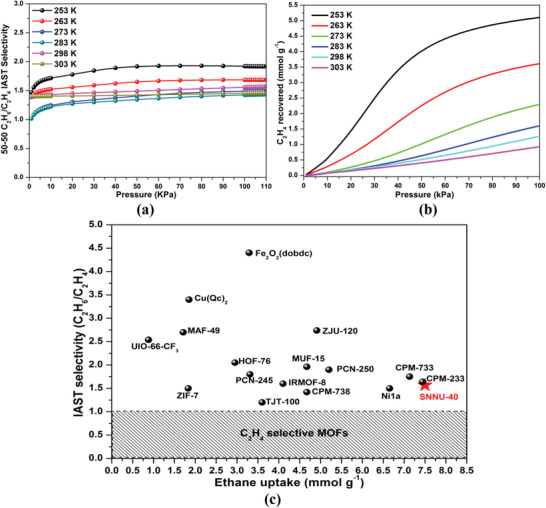
a) The IAST‐calculated selectivities, and separation potential (calculated using Equations (S3)–(S6), Supporting Information) b) for C_2_H_6_/C_2_H_4_ (50/50) mixture of SNNU‐40, and IAST selectivity versus c) single‐component ethane uptake for the select high‐performance ethane‐selective materials reported to date.

To understand the host–guest interactions, the distribution of C_2_H_4_ and C_2_H_6_ in SNNU‐40 at 298 K and 1 bar was studied by GCMC simulation. As shown in **Figure** **4**, the high uptake of C_2_H_6_ in SNNU‐40 should be attributed to the fact that C_2_H_6_ molecules can interact more strongly with the microporous channel walls through C—H⋅⋅⋅O (H⋅⋅⋅O, 2.86–3.98 Å) and C—H⋅⋅⋅*π* (H⋅⋅⋅*π*, 3.75–4.56 Å) interactions. While the planarity of C_2_H_4_ molecules makes them only form weaker interactions with surrounding Lewis basic sites and near pyridine rings (C—H⋅⋅⋅O, 3.20–4.18 Å; C‐H⋅⋅⋅*π*, 4.53–4.98 Å). These results reveal an unusual affinity of SNNU‐40 towards inert C_2_H_6_ instead of unsaturated C_2_H_4_, giving an affinity order of C_2_H_6_ > C_2_H_4_ and resulting in inverse selectivity (i.e., C_2_H_6_‐selective) by SNNU‐40.

**Figure 4:**
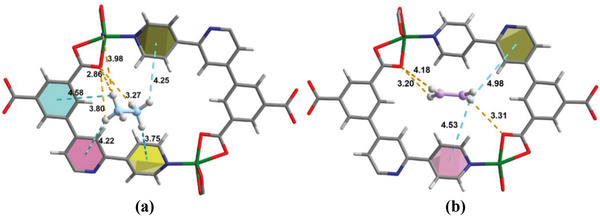
Results of the GCMC simulations showing preferential binding sites between a single adsorbed molecule and SNNU‐40: a) C_2_H_6_ and b) C_2_H_4_.

The isosteric heat of adsorption (*Q*_st_) was studied by the virial model to analyze the adsorption properties. With increasing coverage, the *Q_st_* does not change significantly and the adsorption enthalpy for C_2_H_4_, C_2_H_2_, C_2_H_6_, CO_2_, and CH_4_ were in the range of 18.1−19.4, 25.8−25.3, 18.0−21.4, 20.7−20.4, and 16.1−21.3 kJ mol^−1^, respectively, suggesting uniformity of binding sites (Figure S13, Supporting Information). These values are much lower than those for MOF‐74 series^[^ ^40^ ^]^ and UTSA‐60^[^ ^41^ ^]^ with open metal sites.

Compared to Co/Mg/Ni‐MOF‐74^[^ ^5^, ^40^ ^]^ whose *Q*_st_ decreases as the uptake increases (suggesting a quick saturation of open metal sites), SNNU‐40 has a fairly consistent *Q*_st_ value even at high uptake, suggesting the drastically different uptake behavior from MOFs with open metal sites. These results indicate that the energy required for regeneration of SNNU‐40 will be lower than that of MOF‐74 series^[^ ^40^ ^]^ and Cu‐TDPAT,^[^ ^16^ ^]^ resulting in a significant energy saving. Such exceptionally low binding energy for SNNU‐40 was mainly attributed to the absence of both open metal sites and free —NH_2_ or —OH groups in SNNU‐40. Only the large aromatic sites of SNNU‐40 afford the weak C—H⋅⋅⋅O and C—H⋅⋅⋅*π* binding energy to guest molecules.

Combining the inverse C_2_H_6_/C_2_H_4_ selectivity and high uptake capacity, SNNU‐40 shows potential for C_2_H_6_/C_2_H_4_ separation. Achieving this combination of good selectivity and high capacity is rare in an adsorbent material (Figure 3c; Table S7, Supporting Information). To evaluate the performance of SNNU‐40 in an actual adsorption‐based separation process, breakthrough experiments were performed in which an equimolar C_2_H_6_/C_2_H_4_ mixture was flowed over a packed bed of SNNU‐40. As shown in **Figure** **5**, SNNU‐40 can effectively separate an equimolar mixture of ethylene and ethane. At 1 bar, the breakthrough experiments at three different temperature of 273, 283, and 298 K show that C_2_H_4_ gas was detected from the separation bed first, while no C_2_H_6_ appeared until about 100, 60, and 30 min. SNNU‐40 has good dynamic separation performance around room temperature, suggesting that C_2_H_6_/C_2_H_4_ separation can be cheaply achieved without precisely controlling temperature in industrial settings. At 298 K, the breakthrough experiments at four different flow rates of 2, 4, 6, and 8 mL min^−1^ show that the smaller the flow rate, the better the separation effect (Figure 5b). The separation efficiency under the same temperature at 1 atm is better than that at 2 atm, demonstrating that SNNU‐40 is better suited for removing C_2_H_6_ from C_2_H_4_ at lower pressures. The “rollup” phenomenon can be ascribed to the desorption of initially adsorbed C_2_H_6_ and simultaneous occupation by C_2_H_4_. In the normal industrial environment, the adsorbent should possess good regenerability and structural stability. To ensure the regenerability of SNNU‐40, 50/50 C_2_H_6_/C_2_H_4_ separation cycling experiments were performed at 298 K (Figure 5d). The experimental cycling results indicate that there was no noticeable loss in separation capacity for SNNU‐40 after five cycles. Furthermore, the stability of SNNU‐40 was verified by PXRD after separation cycling experiments (Figure S2, Supporting Information).

**Figure 5:**
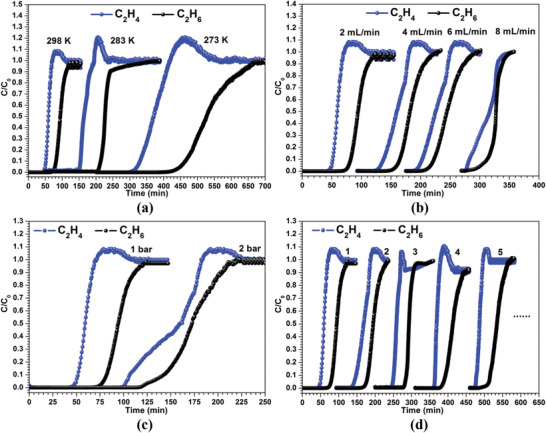
Breakthrough curves of equimolar C_2_H_4_ and C_2_H_6_ mixtures for SNNU‐40: a) variable temperature (273, 283, and 298 K) at 1 atm and 2 mL min^–1^, b) variable velocity (2, 4, 6, and 8 mL min^–1^) at 298 K and 1 atm, c) variable pressure 1 and 2 atm at 298 K and 2 mL min^–1^, and d) the cycling experiments of 2 mL min^–1^ at 298 K and 1 atm.

SNNU‐40 can also be used to remove C_2_H_4_ for storage and transport of fruits, vegetables, and flowers.^[^ ^9^, ^10^ ^]^ To investigate the selective adsorption of ethylene over other gases, the breakthrough experiments were performed in which an equimolar ethylene/dry air mixture was flowed over a packed bed of SNNU‐40 with a flow rate of 2 mL min^–1^. As shown in **Figure** **6**a, C_2_H_4_ was the last to elute from the bed, demonstrating that SNNU‐40 has good selectivity to C_2_H_4_.

**Figure 6:**
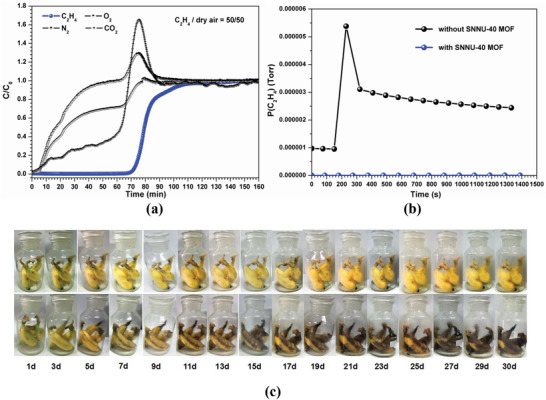
a) Breakthrough curves of equimolar C_2_H_4_ and dry air mixtures for SNNU‐40, the freshness preservation experiments with or without SNNU‐40: b) the ethylene partial pressure in the jars after 30 days, and c) the banana changing process recorded by taking pictures with different time intervals.

Further, to demonstrate the effectiveness of ethylene abatement and adsorption by SNNU‐40 in freshness preservation, we compared the change of bananas with and without SNNU‐40. Two groups of green and fresh bananas were selected and placed in two 1 L sealed glass jars, one with 30 mg activated SNNU‐40, one without. Each jar was filled with nitrogen to prevent oxidation and interference by other gases. Photographs were taken every day to monitor the state of bananas. As shown in Figure 6c, the bananas without SNNU‐40 began to have dark spots on the fifth day, had a large area of dark spots on the ninth day, and completely went bad on the 15th day. While the group with SNNU‐40 to capture ethylene showed no signs of spoilage on the 30th day. The use of SNNU‐40 as ethylene adsorbent has the following advantage: the use level (3 g/50 kg) in our simulated experiment is much less than dried perlite that has been fully absorbed potassium permanganate on the market (1 g/5 kg). This showed that SNNU‐40 has long‐term preservation effect on banana, further proving it can be used for extending quality, prolonging freshness and enhancing flavor of fresh fruits, vegetables, and flowers.

In conclusion, we report here a single‐walled porous MOF SNNU‐40 without open metal sites but exhibiting exceptionally high ethylene and ethane storage capacity, which surpass all MOF adsorbents under 273 K and 1 bar condition, even though its heat of adsorption (*Q*_st_) is relatively low. The combination of low *Q*_st_ and high‐capacity uptake could bring a distinct economic advantage because of the significantly reduced energy consumption for low‐temperature activation and regeneration of adsorbents. SNNU‐40 also shows an ethane‐selective ethane–ethylene separation performance with top‐level breakthrough times. Also important is that we demonstrate the application of SNNU‐40 for direct air capture of excess ethylene for the purpose of preserving fruits, which helps to address the key industry challenges of food security, profitability, and global food waste.

## Experimental Section

The experimental details are listed in the Supporting Information.

## Conflict of Interest

The authors declare no conflict of interest.

## Supporting information

Supporting Information

## Data Availability

The data that supports the findings of this study are available in the supplementary material of this article.
