# Supplementary material for: Ultrahigh‐Uptake Capacity‐Enabled Gas Separation and Fruit Preservation by a New Single‐Walled Nickel–Organic Framework
Source: Adv Sci (Weinh). 2021 May 1;8(12):2003141. doi: 10.1002/advs.202003141 (PMC8224448; doi:10.1002/advs.202003141)
Supplement: Supplementary file 1 — Supporting Information [file ADVS-8-2003141-s001.pdf]

WILEY-VCH

© 2020 Wiley-VCH GmbH

ADVANCED SCIENCE
Open Access

Supporting Information

for *Adv. Sci.,* DOI: 10.1002/advs.202003141

Ultra-High Uptake Capacity-Enabled Gas Separation and Fruit Preservation by a New Single-Walled Nickel-Organic Framework

Yong-Peng Li, Yong-Ni Zhao, Shu-Ni Li, Da-Qiang Yuan
, Yu-Cheng Jiang, Xianhui Bu, Man-Cheng Hu, and Quan-Guo Zhai**

Supporting Information

Ultra-High Uptake Capacity-Enabled Gas Separation and Fruit Preservation by a New Single-Walled Nickel-Organic Framework

Yong-Peng Li, Yong-Ni Zhao, Shu-Ni Li, Da-Qiang Yuan, Yu-Cheng Jiang, Xianhui Bu, Man-Cheng Hu, Quan-Guo Zhai**

Section S1: Experimental and measurements

Materials and Methods

All chemicals were obtained from commercial sources and used without further purification. Thermogravimetric analyses (TGA) were carried out on a NETSCHZ STA-449C thermal analyzer at a ramp rate of 5 °C min^{-1} in nitrogen atmosphere for SNNU-40. Powder X-ray diffraction (PXRD) studies were carried out with a Japan Rigaku D/Max2550VB+/PC diffractometer equipped with Cu Kα radiation (λ = 1.5406 Å). Specific surface area measurements at 77 K were carried out using a Micromeritics ASAP 2020 adsorption porosimeter (Micromeritics Instrument Corp., USA) with N_2 gas. Samples were outgassed in vacuum at 50 ^{o}C for 10 h to remove all guest molecules prior to the test. The pore textural properties including the Brunauer−Emmet−Teller (BET) surface area was obtained from the N_2 adsorption and desorption isotherms.

Synthesis of H_2DBPT

According to literature[S1], a mixture of 3,5-dimethyl-benzaldehyde (2.68 g, 20 mmol), 4-acetylpyridine (5.04 g, 40 mmol) were added in the cold ethanol and stirred for 30 minutes, and next solid KOH (1.6 g, 40 mmol) was ground and added in the above reaction mixture. After about 2 h, 60 mL ammonia was slowly dropped into the above solution and then reflux for 24 hours. Upon cooling, the white precipitate were filtered from the solution, washed with cold ethanol and dried. Then 5 mL HNO_3 (ca. 65%) and 1 mL H_2O were mixed with the above product (400 mg), and the mixture were moved to a Teflon-lined stainless vessel (25 mL) and heated at 180 ^{o}C for 24 h. The vessel was cooled slowly down to room temperature

at 1.5 ^{o}C/h, affording light yellow crystals with the yield of 57%. The crystal has the same unit cell parameters as the literature.

Synthesis of SNNU-40

A mixture of $Ni(NO_3)_2·6H_2O$ (0.014 g, 0.05 mmol), H_2DBPT (0.020 g, 0.05 mmol) and 3 mL DMA/DMI (v/v = 2:1) in a 20 mL vial, while stirring at room temperature. After a few minutes, the mixed solution was sealed, which was heated at 120 ^{o}C for 8 days and then it was slowly cooled to room temperature. Green octahedral crystals of as-synthesized SNNU-40 were collected in 30% yield based on Ni(II). The crystal photos, powder XRD and FT-IR spectra of SNNU-40 have been provided in Figures S2−S5, which clearly confirmed the phase purity of as-synthesized products.

X-ray Crystallographic Determination

Single-crystal X-ray analysis was performed on a Bruker Smart APEX II CCD area diffractometer using graphite-monochromated Mo Kα radiation (λ = 0.71073 Å). The SADABS program was used for absorption correction. The structure was solved by direct methods, and the structure refinements were based on $|F|^2$ with anisotropic displacement using *SHELXTL*[S2-4]. All non-hydrogen atoms in the framework were refined with anisotropic displacement parameters. Crystal data as well as details of data collection and refinements were summarized in Table S1-S2 (CCDC # 2012688).

Gas Adsorption

Gas sorption isotherms were measured on a Micromeritics ASAP 2020 HD88 surface-area and pore-size analyzer up to 1 atm of gas pressure by the static volumetric method. All gases

used were of 99.99% purity. The gas sorption isotherms for N_2 and H_2 were measured at 77 K with liquid nitrogen. The gas sorption isotherms for C_2H_2, CO_2 and CH_4 were measured at 273 K and 298 K, C_2H_4 and C_2H_6 were measured at 253 K, 263 K, 273 K, 283 K, 298 K and 303 K through the control thermometer of ethanol cycle, respectively.

Isosteric Analysis of the Heat of Adsorption

To extract the coverage-dependent isosteric heat of adsorption, the data were modeled with a virial-type expression composed of parameters a_i and b_i that are independent of temperature:

$$\ln P = \ln N + \frac{1}{T}\sum_{i=0}^{m} a_i N^i + \sum_{i=0}^{n} b_i N^i \qquad (1)$$

where P is pressure, N is the amount adsorbed (or uptake), T is temperature, and m and n determine the number of terms required to adequately describe the isotherm.

$$Q_{st} = - \mathrm{R} \sum_{i=0}^{m} a_i N^i \qquad (2)$$

where R is the universal gas constant. The coverage dependencies of Q_{st} calculated from fitting the 273 K and 298 K (under the pressure range from 0-1 bar) data are presented graphically in Figures S14 for SNNU-40.

Selectivity Prediction for Binary Mixture Adsorption

Ideal adsorbed solution theory (IAST) was used to predict binary mixture adsorption from the experimental pure-gas isotherms. To perform the integrations required by IAST, the single component isotherms should be fitted by a proper model. The Dual-site Langmuir-Freundlich (LF) equation[S5] was found to the best fit to the experimental pure isotherms for C_2H_6, C_2H_4, C_2H_2, CO_2, and CH_4 of SNNU-40.

$$q = q_{m1} * \frac{b_1 * p^{1/n_1}}{1 + b_1 * p^{1/n_1}} + q_{m2} * \frac{b_2 * p^{1/n_2}}{1 + b_2 * p^{1/n_2}} \quad (3)$$

where p is the pressure of the bulk gas at equilibrium with the adsorbed phase (kPa), q is the adsorbed amount per mass of adsorbent (mmol g^{-1}), q_m is the saturation capacities of site (mmol g^{-1}), b is the affinity coefficients of site (1/kPa), and n represent the deviations from an ideal homogeneous surface. Table S6 presents the fitting parameters of DSLF equation as well as the correlation coefficients (R^2).

It should be noted that: q_{m2} = 0, b_2 = 0 and $1/n_2$ = 1, it is Langmuir-Freundlich model;

Based on the above equation parameters of pure gas adsorption, we used the IAST model to investigate the separation of C_2H_6/C_2H_4, C_2H_4/CO_2, C_2H_2/C_2H_4, C_2H_4/CH_4 and C_2H_4/N_2 and CO_2/CH_4 in compound SNNU-40, the adsorption selectivity is defined by

$$s_{A/B} = \frac{x_A / y_A}{x_B / y_B} \quad (4)$$

Where x_i and y_i are the mole fractions of component i (i = A and B) in the adsorbed and bulk phases, respectively. Note that in the Henry regime $S_{A/B}$ is identical to the ratio of the Henry constants of the two species.

Separation Potential

The separation potential (ΔQ)[S5] is a combined metric, which considering both uptake capacity and selectivity. It is defined to quantify mixture separations in fixed bed adsorber. For a C_2H_6/C_2H_4 mixture with mole fractions y_{C2H6}, and $y_{C2H4}=1-y_{C2H6}$, the gravimetric separation potential ΔQ, is calculated from IAST using the formula

$$\Delta Q = q_{C_2H_6}\frac{y_{C_2H_4}}{1-y_{C_2H_4}} - q_{C_2H_4} \qquad (5)$$

where q_{C2H6} and q_{C2H4} are C_2H_6 and C_2H_4 uptake in the mixture, respectively, which are calculated based on IAST theory. For 50/50 mixture, the formula (5) can be simplified as

$$\Delta Q = q_{C_2H_6} - q_{C_2H_4} \qquad (6)$$

The physical significance of ΔQ is that it represents the maximum amount of pure C_2H_4 that can be recovered during the adsorption phase of fixed bed separations.

Breakthrough Separation Experiments and Procedures

The breakthrough experiments were carried out in dynamic gas breakthrough equipment (The equipment built by cooperating with instrument manufacturers according to the basic principles reported in the literature). A stainless-steel column with a length of 170.1 mm and an internal diameter of 4.1 mm was used for sample packing. Activated crystalline sample (1.56 g) was packed into the column and absorbent cotton was used to seal the ends of the steel column. The column was placed in a circulating jacket connected to a thermostatic bath (temperature ranged from 233 or 398 K). The mixed gas flow and pressure were controlled by using a pressure controller valve and a mass flow controller. Outlet effluent from the column was continuously monitored using gas analytical mass spectrometer (Hiden, HPR-20 R&D). The column packed with sample was firstly purged with He flow (30 mL/min) for 6 h at room temperature 298 K. The gas flows were controlled at the inlet by a mass flow meter, and prior to every breakthrough experiment, we activated the sample by flushing the adsorption bed with helium gas for 1 hour at 298 K. Subsequently, the column was allowed to equilibrate at the measurement rate before we switched the gas flow.

Stability Tests

Crystalline samples of SNNU-40 were exposed to the different solvents and moisture with the humidity of 80% at 298 K for 2 days. This humidity condition was achieved by using a constant temperature and humidity incubator. After the exposure, the sample was then characterized by PXRD measurements.

Grand Canonical Monte Carlo (GCMC) Simulation

All simulations were done in the Material Studio 8.0. The unit cell of 1 × 1 × 1 was used as the simulation box. The individual C_2H_4 molecules and the framework were considered to be rigid. Simulation was performed for a model that includes electrostatic and Lennard-Jones potential among the atoms in the system. Atomic partial charges derived from QEq method. All parameters for the atoms were modeled with the universal forcefield (UFF) embedded in the MS modeling package. The cut-off radius used for the Lennard-Jones interactions is 12.5 Å, and the electrostatic interactions were treated by using the Ewald summation. The preferred locations of adsorbed gas molecule were simulated by the fixed loading task in the sorption model. For each run, the 5×10^6 maximum loading steps, 5×10^6 production steps were employed.

Banana Freshness Preservation Experiment

Two groups of green and fresh bananas (about 500 g) were selected and placed in two 1 L sealed glass jars, one with 30 mg activated SNNU-40, one without. Each jar was filled with nitrogen to prevent oxidation and interference by other gases. Two jars were placed in an

incubator at room temperature. Photographs were taken every day to monitor the state of bananas.

Statistical Analysis

1. The structure obtained from single-crystal X-ray analysis was solved by SHELXTL, further cif document was imported into the Diamond software. Finally, the detailed structure analysis and corresponding images were obtained.

2. The Heat of Adsorption, Selectivity, and Separation Potential were obtained by the above corresponding part formula. Downstream data analyses including statistical analysis, plotting, and data fitting were performed with Excel and Origin.

Section S2: Tables of single crystal information for SNNU-40

Table S1. Crystal data and structure refinements for SNNU-40.

**Compound**	**SNNU-40**
Empirical formula	$C_{23}H_{13}N_3O_4Ni$
Formula weight	454.07
Crystal system	Tetragonal
Space group	*P*4(2)/*nmc*
a (Å)	22.608(2)
b (Å)	22.608(2)
c (Å)	21.117(2)
α (deg)	90
β (deg)	90
γ (deg)	90
V (Å^3)	10793.5(17)
Z	8
D_{calcd}(Mg·m^{-3})	0.559
μ(mm^{-1})	0.373
F(000)	1856
θ for data collection (deg)	2.23 to 25.02
Reflections collected/unique	43694/4974
R(int)	0.1493
parameters	184
GOF on F^2	1.086
R_1^a, wR_2 [I > 2δ (I)]	0.0971, 0.2325
R_1, wR_2 (all data)	0.1310, 0.2398
ρ_{fin}(max/min) (e·Å^{-3})	2.633/-0.507

[a] $R_1 = \Sigma||F_o|-|F_c|| / \Sigma|F_o|$. [b] $wR_2 = [\Sigma w(F_o^2-F_c^2)^2 / \Sigma w(F_o^2)^2]^{1/2}$.

Table S2. Selected bond lengths (Å) and angles (o) for SNNU-40.

Ni(1)-N(2a)	2.021(4)	O(2)-Ni(1)-O(2c)	158.36(18)
Ni(1)-N(2b)	2.021(4)	N(2a)-Ni(1)-O(1)	91.04(13)
Ni(1)-O(2)	2.071(3)	N(2b)-Ni(1)-O(1)	159.79(15)
Ni(1)-O(2c)	2.071(3)	O(2)-Ni(1)-O(1)	61.84(11)
Ni(1)-O(1)	2.190(3)	O(2c)-Ni(1)-O(1)	101.75(11)
Ni(1)-O(1c)	2.190(3)	N(2a)-Ni(1)-O(1c)	159.79(15)
N(2a)-Ni(1)-N(2b)	96.0(2)	N(2b)-Ni(1)-O(1c)	91.04(13)
N(2a)-Ni(1)-O(2)	95.94(15)	O(2)-Ni(1)-O(1c)	101.75(11)
N(2b)-Ni(1)-O(2)	98.50(15)	O(2c)-Ni(1)-O(1c)	61.84(11)
N(2a)-Ni(1)-O(2c)	98.50(15)	O(1)-Ni(1)-O(1c)	88.78(15)
N(2b)-Ni(1)-O(2c)	95.94(15)		

Symmetry codes: a) - y + 1/2, x, z - 1/2; b) x + 1/2, - y, - z + 1; c) y + 1/2, x - 1/2, - z + 1/2; d) - x + 1/2, y, z; e) y, - x + 1/2, z + 1/2.

Section S3: Crystal structures for SNNU-40

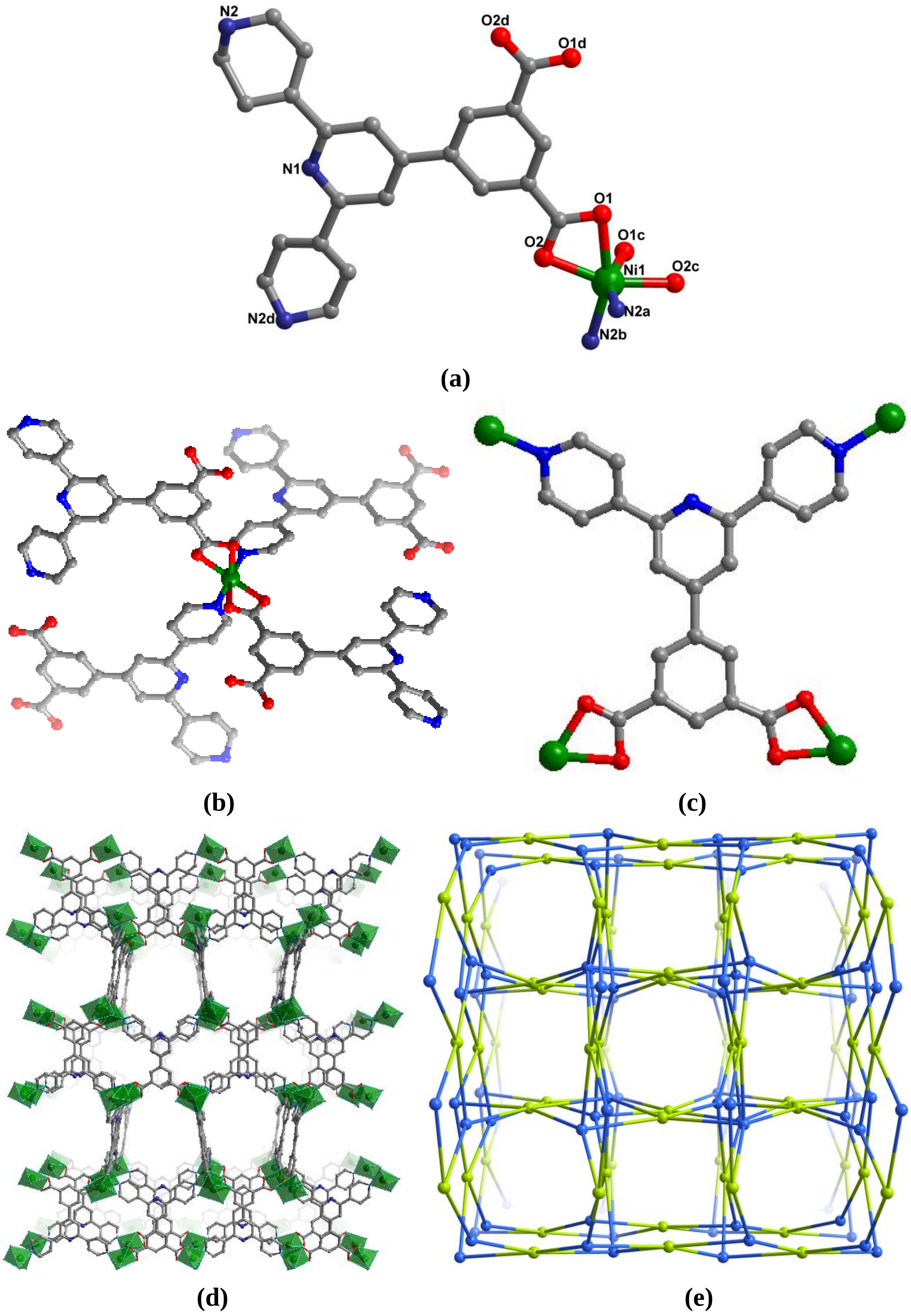

Figure S1. The coordination environment of mononuclear Ni(II) in SNNU-40 (a); The distribution of H_2DBPT ligands around the mononuclear Ni(II) (b) and the coordination modes of H_2BPDC ligand (c); 3D structure viewed along *b* axis (d); and 4,4-connected *PtS* topology (e).

Section S4: Structural characterization

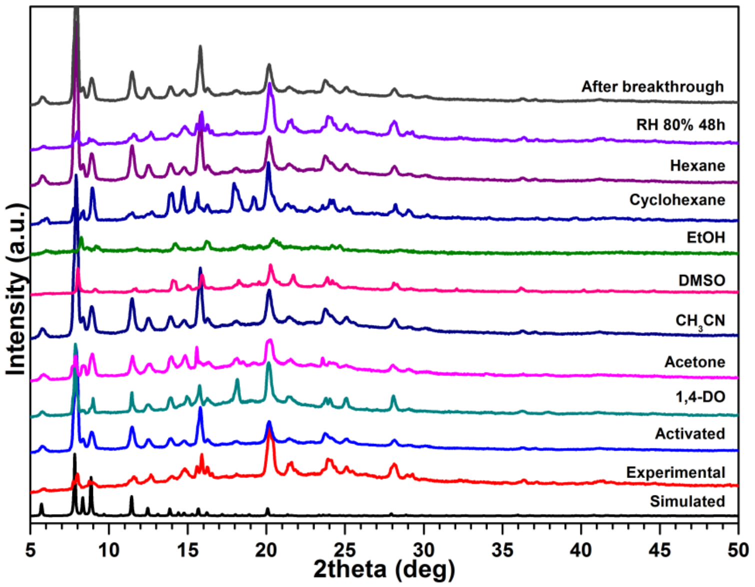

Figure S2. PXRD patterns for SNNU-40: the simulated pattern from X-ray single-crystal data, as-synthesized sample, after the treatments with different solvents and under 80% moisture at room temperature for 48h, and after breakthrough experiments.

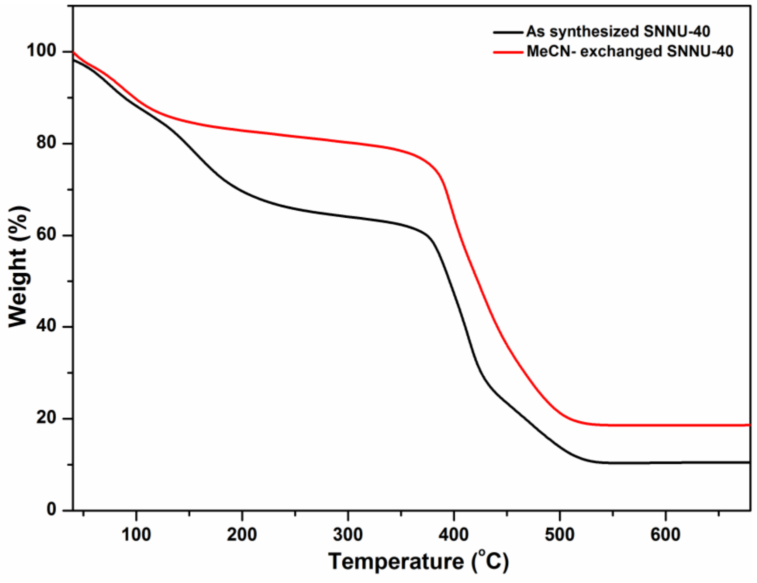

Figure S3. TGA curves of as-synthesized (black) and acetonitrile-exchanged SNNU-40 (red).

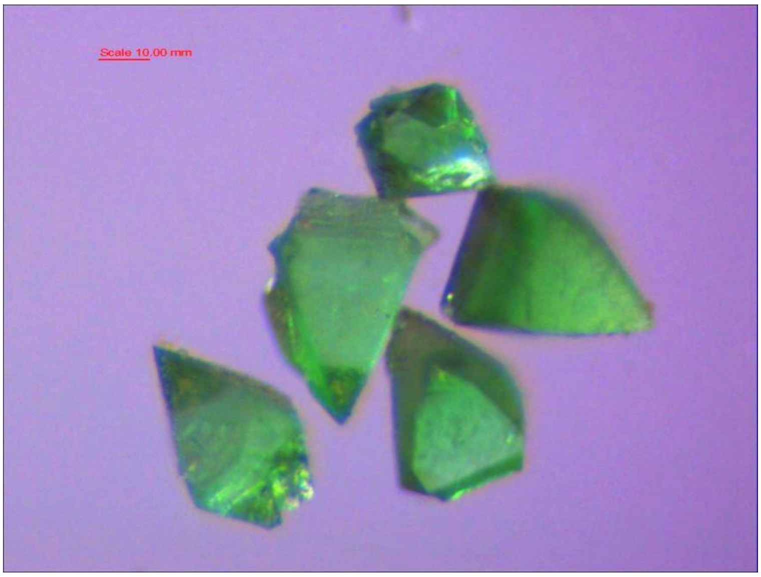

Figure S4. Optical microscope image of SNNU-40.

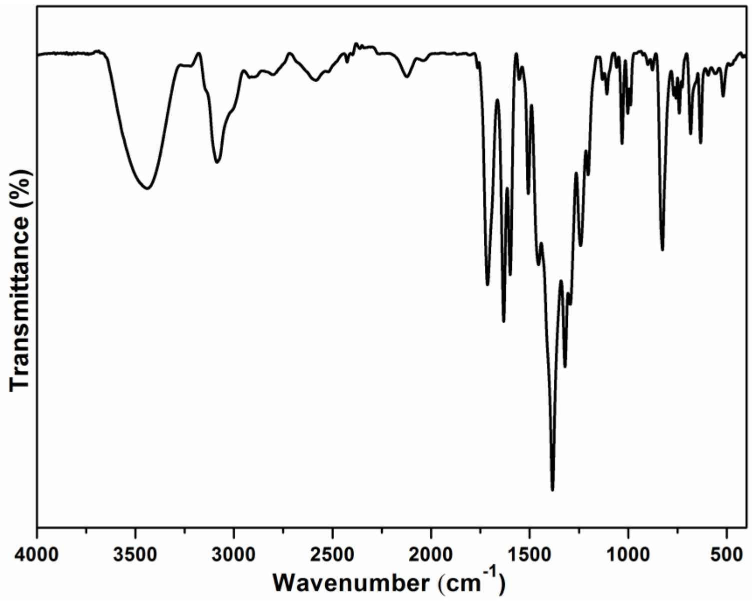

(a)

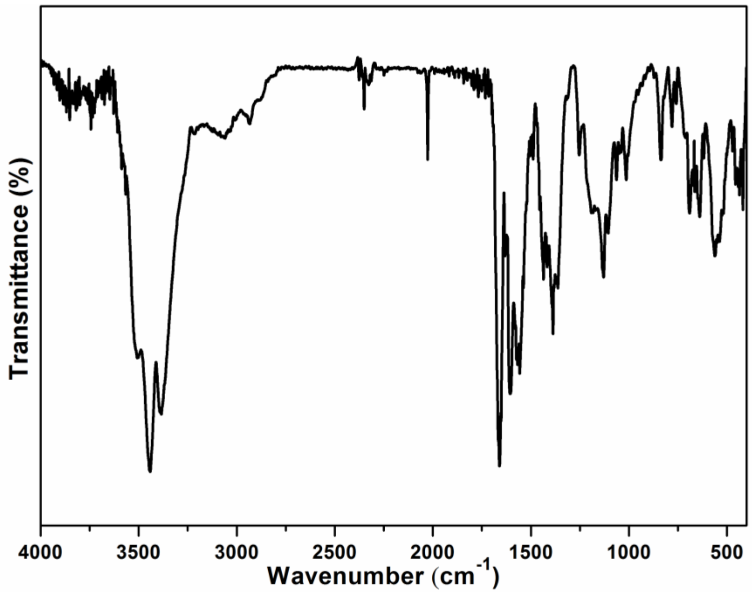

(b)

Figure S5. FT-IR spectra for H_2DBPT (a), and SNNU-40 (b).

Section S5: Gas adsorption measurements for SNNU-40

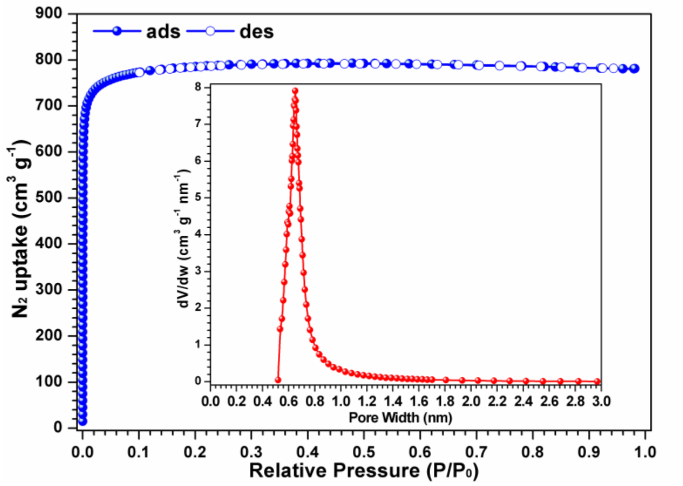

Figure S6. N_2 isotherms at 77 K (inset: pore size distribution).

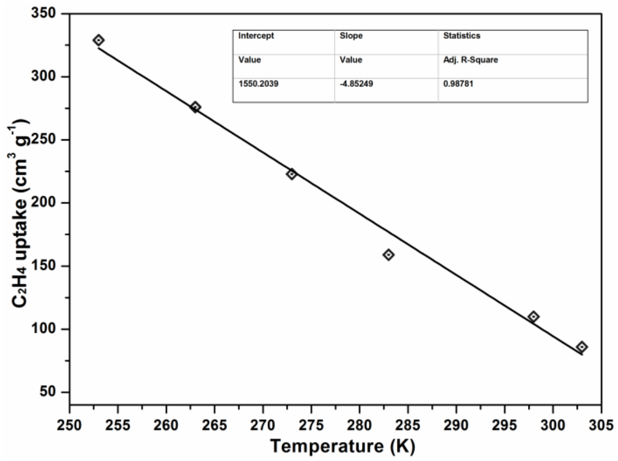

(a)

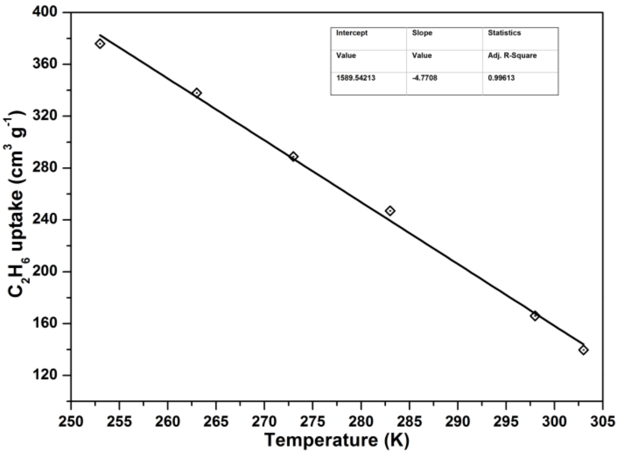

(b)

Figure S7. Linear relation between the temperature (253-303 K) and gas uptake amount: C_2H_4 (a) and C_2H_6 (b).

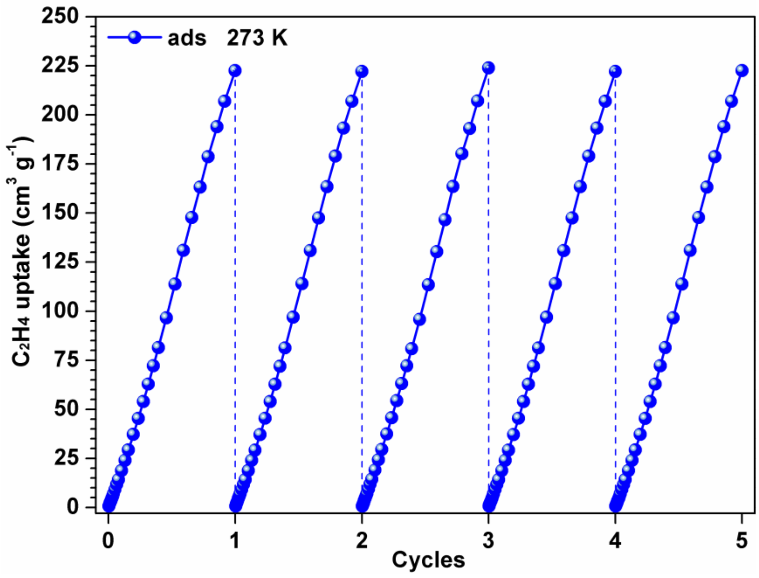

(a)

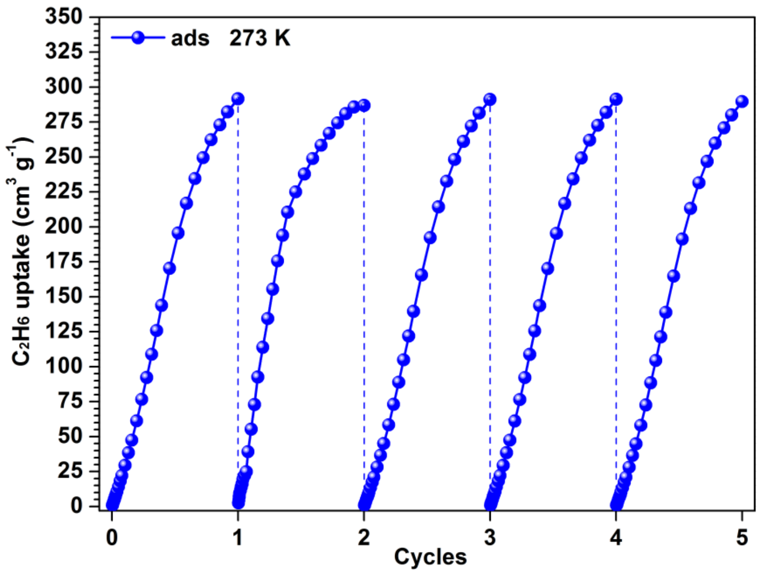

(b)

Figure S8. Cycles of C_2H_4 (a) and C_2H_6 (b) adsorption at 273 K.

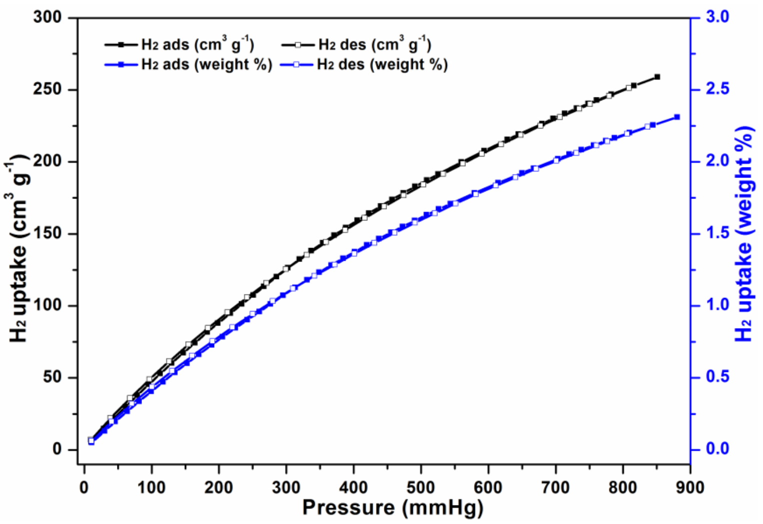
H2 ads (cm3 g-1)
H2 des (cm3 g-1)
H2 ads (weight %)
H2 des (weight %)
H2 uptake (cm3 g-1)
H2 uptake (weight %)
Pressure (mmHg)

(a)

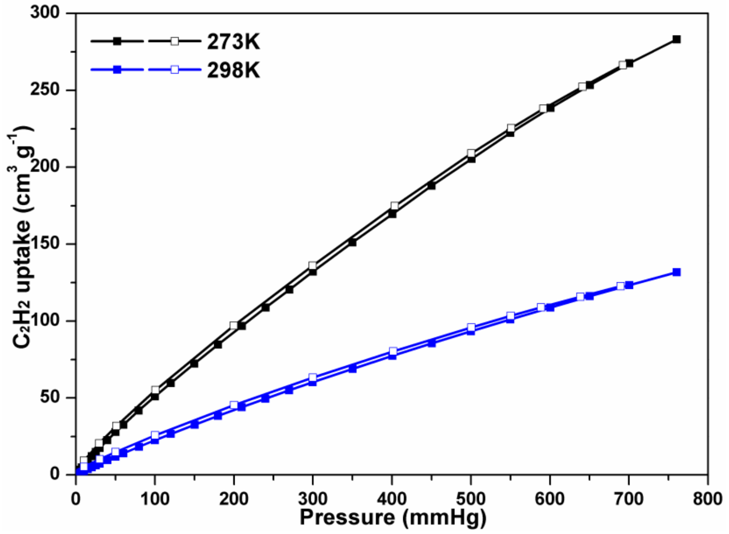
273K
298K
C2H2 uptake (cm3 g-1)
Pressure (mmHg)

(b)

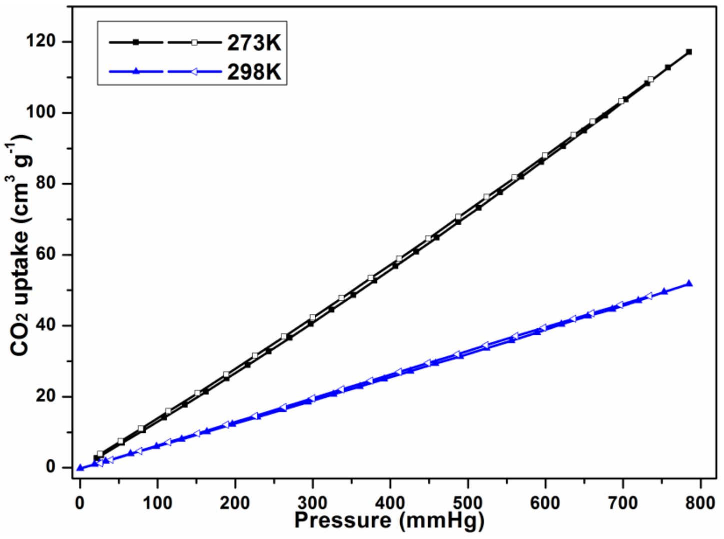
273K
298K
CO2 uptake (cm3 g-1)
Pressure (mmHg)

(c)

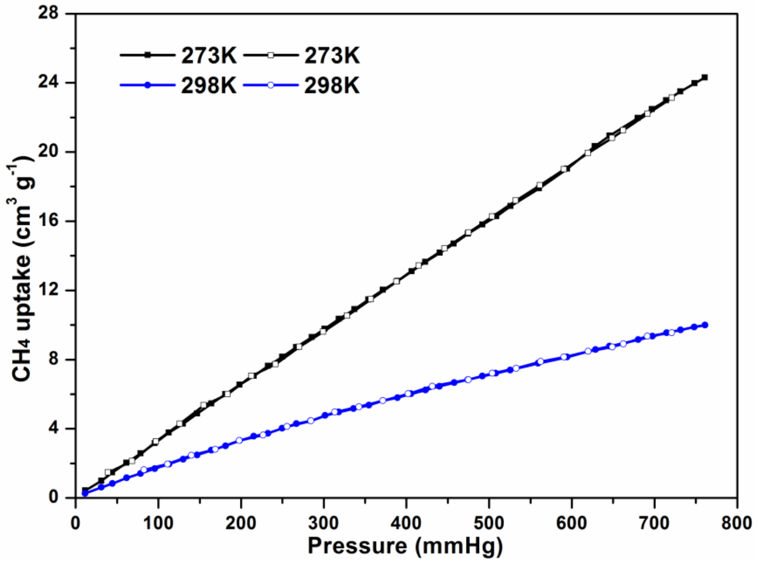

(d)

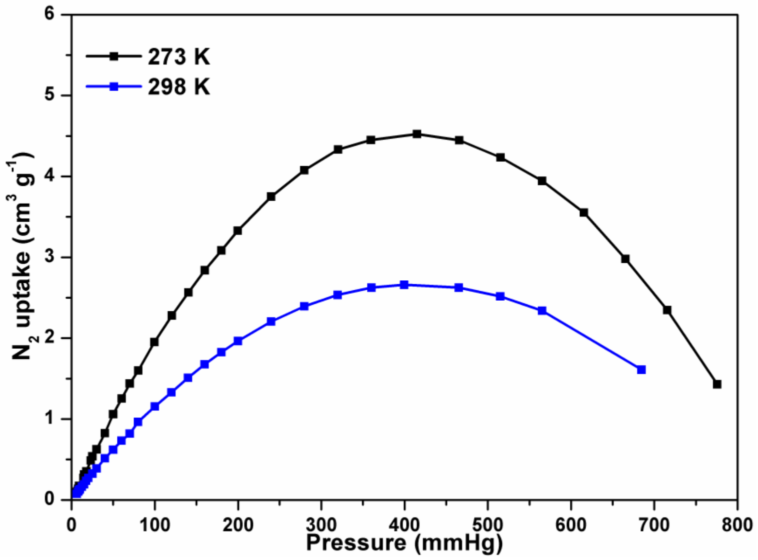

(e)

Figure S9. H_2 (a), C_2H_2 (b), CO_2 (c), CH_4 (d) and N_2 (e) adsorption and desorption isotherms of SNNU-40 (Solid and open symbols indicate adsorption and desorption isotherms, respectively).

Table S3. Summary of top-level C_2H_4 uptake MOF materials (273K, 298 K and 1 bar).

Material	BET (Langmuir) [m^2/g]	Pore volume [cm^3/g]	C_2H_4 uptake [cm^3/g] 273 K	C_2H_4 uptake [cm^3/g] 298 K	Q_{st} (C_2H_4) [kJ/ mol]	Ref
**SNNU-40**	**2233.8 (3484.7)**	**1.22**	**224**	**110**	**20.1**	**This work**
CPM-223-tppy	1599	-	221	164	22.6	[S6]
Cu-TDPAT	1938 (2608)	0.93	218	164	49.5	[S7]
ZJU-11	2743 (2531)	1.01	217	157	36.1	[S8]
PCN-16	(2810)	1.00	210	160^b	-	[S9, 10]
NEM-4	2278	0.92	208	164^c	-	[S11]
SNNU-65-Cu-Sc	2089.2	1.14	206	122	61.4	[S12]
NOTT-101	2316 (2929)	1.048	205	145^b	-	[S9, 13]
NOTT-102	(3590)	1.28	200	128^b	-	[S9, 13]
CPM-733	1328	-	190	143	22.5	[S6]
CPM-233	1598	-	189	146	26.7	[S6]
Cu-BTC	2139	0.76	185	165^b	26.5	[S9]
MgMOF-74	(1668)	0.61	180	165^b	42-26.0	[S9, 14]
Co-MOF-74	(1449)	0.51	175	165^b	41.0-25.0	[S9]
UMCM-150	(3330)	1.21	175	120^b	-	[S9]
ZJU-60	1627 (2394)	0.867	159	132^b	21.4	[S15]
MOF-505	1139 (1703)	0.609	142	113^b	-	[S9, 16]
LIFM-26	1513	0.59	142	100	45	[S17]
PCN-250	1470	0.564/0.506	112	94.5	21.1-25.3	[S18]
UiO-66	1014 (1439)	0.40	80	53	18–28	[S19]
TJT-100	890 (1077)	-	98	75	25	[S20]
UTSA-33	660.0 (1024.3)	0.367	85	60^b	31	[S21]
HOF-76	1121	0.40	78	37B	20.5	[S22]
FJI-C4	690 (781)	0.27	70.1	61	33.1−42.6	[S23]
$Cu(Qc)_2$	240 (290)	0.11	35	18	25.4	[S24]

The calculations are based on the sorption data at 296 K (b) and 295 K (c).

Table S4. Summary of top-level C_2H_6 uptake MOF materials (273K, 298 K and 1 bar).

Material	BET(Langmuir) [m²/g]	Pore volume [cm³/g]	C_2H_6 uptake [cm³/g]	C_2H_6 uptake [cm³/g]	Q_{st} (C_2H_4) [kJ/ mol]	Ref
			273 K	298 K		
**SNNU-40**	**2233 (3484)**	**1.22**	**289**	**169**	**20.1**	**This work**
NOTT-102	(3590)	1.28	245	130[b]	-	[S9, 13]
PCN-16	(2810)	1.00	235	155[b]	-	[S9-10]
NOTT-101	2316 (2929)	1.048	230	163[b]	-	[S9, 13]
ZJU-11	2743 (2531)	1.01	230	154	27.0	[S25]
Cu-TDPAT	1938 (2608)	0.93	218	154	30.2	[S11]
NEM-4	2278	0.92	214	172[c]	-	[S26]
UMCM-150	(3330)	1.21	208	110[b]	-	[S9]
CPM-223-tppy	1599	-	204	161	25.0	[S6]
CPM-733	1328	-	186	160	23.4	[S6]
Cu-BTC	2139	0.76	180	138[b]	31.2	[S9]
CPM-233	1598	-	178	166	27.3	[S6]
ZJU-60	1627(2394)	0.87	178	136	19.8	[S15]
Mg-MOF-74	(1668)	0.61	175	145[b]	29.0	[S9, 14]
Co-MOF-74	(1449)	0.51	165	144[b]	29.0	[S9]
PCN-250	1470	0.564/0.506	136	117	23.6-27.8	[S18]
TJT-100	890 (1077)	-	105	81	29	[S20]
Cu(Qc)2	240 (290)	0.11	47	42	29	[S24]
UTSA-33	660.0 (1024.3)	0.367	25	12[b]	32	[S21]
FJI-C4	690 (781)	0.27	73	66	32.7−40.9	[S23]
HOF-76	1121	0.40	84	66[b]	22.8	[S22]

The calculations are based on the sorption data at 296 K (b) and 295 K (c).

Section S6: Physical parameters for selected gas molecules

Table S5. Physicochemical characteristics of selected gas molecules.

Gas	Boiling point (K)	Kinetic diameter (Å)	Polarizability $\times 10^{25}/cm^3$	Quadruple moment $\times 10^{26}$/esu cm^2
C_2H_4	169.42	4.163	42.52	1.5
C_2H_6	184.55	4.443	44.3-44.7	0.65
C_2H_2	188.40	3.3	33.3-39.3	-
CO_2	216.55	3.3	29.11	4.3
CH_4	111.66	3.758	25.93	0
N_2	77.35	3.64-3.80	17.403	1.52
O_2	90.17	3.467	15.812	0.39

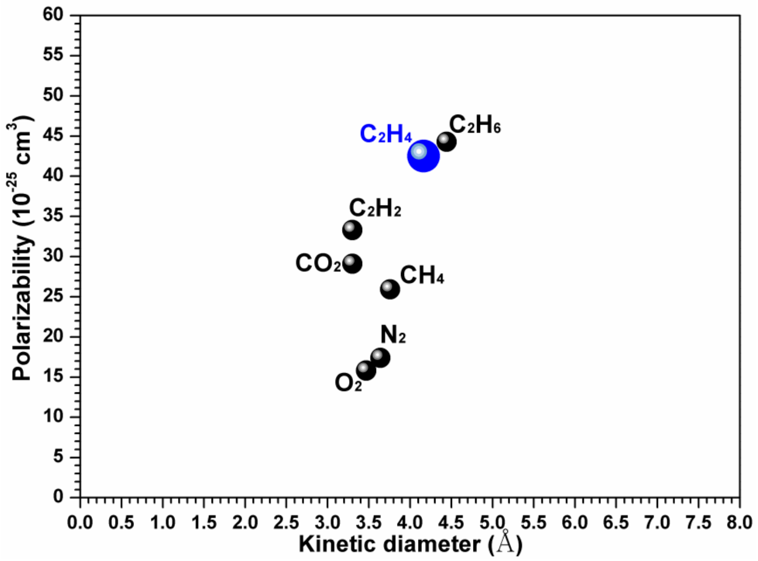

Figure S10. Physical parameters of C_2H_4 and the main impurities.

Section S7: IAST selectivity for SNNU-40

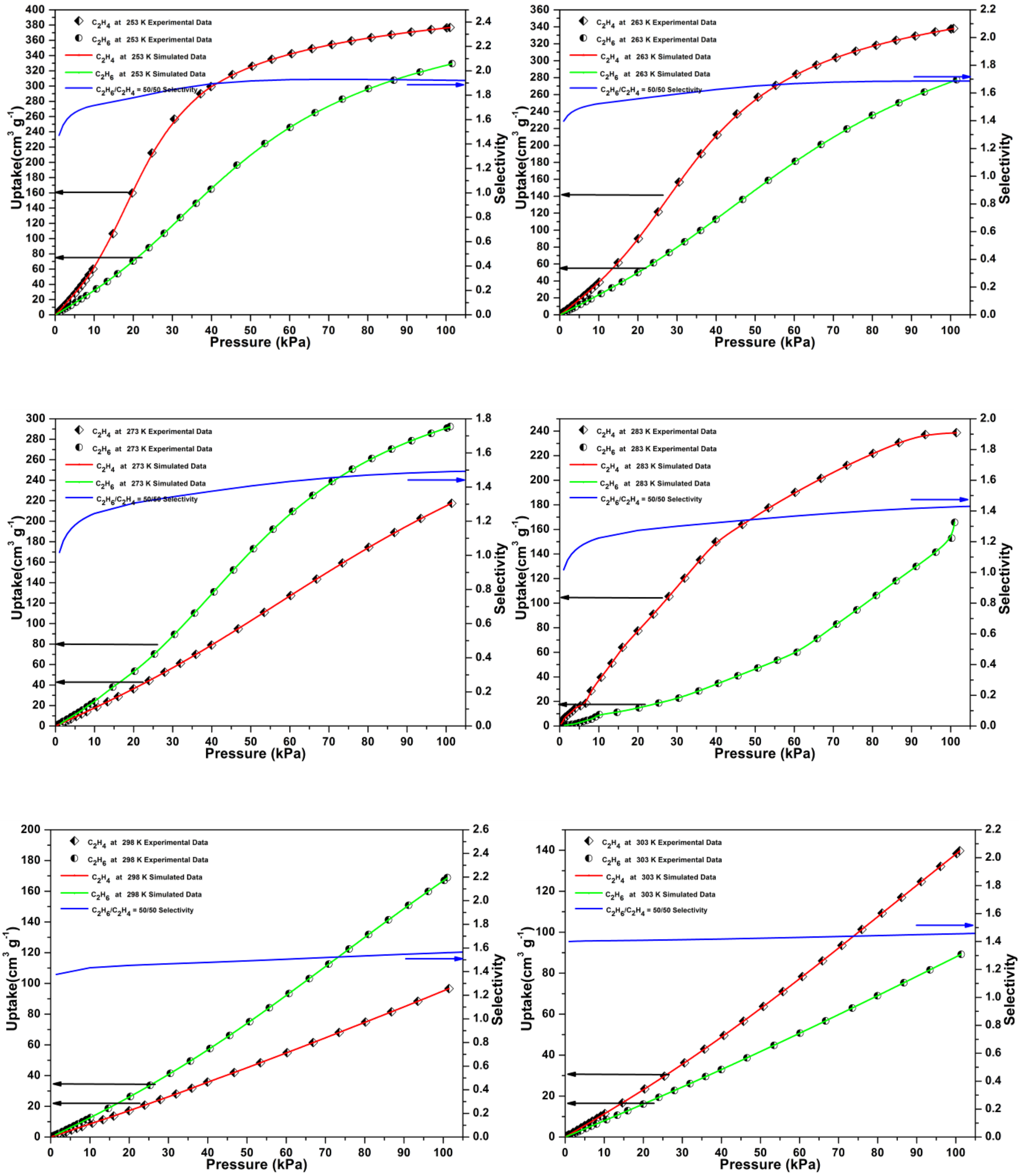

Figure S11. Comparison of experimental isotherms and simulated isotherms (Left Y axis), and mixture adsorption selectivity predicted by IAST (Right Y axis) of SNNU-40 for equimolar C_2H_6/C_2H_4 mixture at different temperatures.

Table S6. The fitting parameters of DSLF model and the corresponding correlation coefficients.

Temperature (K)	Gases	q_{m1} (mmol g^{-1})	b_1 (kPa^{-1})	$1/n_1$	q_{m2} (mmol g^{-1})	b_2 (kPa^{-1})	$1/n_2$	R^2
**253**	C_2H_6	11.7901	0.0188	1.1286	7.7039	1.0093×10^{-5}	3.7085	0.9999
	C_2H_4	8.3747	2.0840×10^{-5}	2.7933	12.8742	0.0105	1.0396	0.9999
**263**	C_2H_6	11.3048	0.0131	1.0960	7.7347	9.8079×10^{-6}	3.2345	0.9999
	C_2H_4	10.4284	0.0095	1.0504	8.9489	1.4394×10^{-5}	2.6445	0.9999
**273**	C_2H_6	7.5696	2.9768×10^{-6}	3.1979	10.7057	0.0080	1.1279	0.9999
	C_2H_4	12.4648	0.0061	1.0246	8.4937	1.2730×10^{-5}	2.4772	0.9999
**283**	C_2H_6	7.6207	4.3398×10^{-6}	2.8912	9.3334	0.0074	1.1033	0.9998
	C_2H_4	15.6033	0.0039	1.0064	8.1271	1.8393	2.2309	0.9999
**298**	C_2H_6	6.1967	5.3470×10^{-6}	2.5688	25.7916	25.7916	1.02690	0.9999
	C_2H_4	224.1931	9.8221	1.1389	0.1650	0.1014	0.9263	0.9999
**303**	C_2H_6	5.6848	8.5824×10^{-6}	2.3594	36.4120	0.0014	1.0015	0.9998
	C_2H_4	8.0782	9.7317×10^{-6}	2.0782	17.4777	0.0020	0.9990	0.9999

Table S7. Summary of C_2H_6 and C_2H_4 uptake properties for ethane-selective MOFs (~ 1 bar and ~ 298 K, 50/50 C_2H_6/C_2H_4 mixture).

Material	C_2H_6 uptake [cm^3/g]	C_2H_4 uptake [cm^3/g]	C_2H_6 /C_2H_4 selectivity	Separation potential (mmol/g)	Ref
**SNNU-40**	**169**	**110**	**1.57**	**1.27**	**This work**
Fe_2O_2(dobdc)	74	57	4.4	1.9	[S27]
$Cu(Qc)_2$	42	18	3.41	0.85	[S24]
ZJU-120 [a]	110	88	2.74	2.09	[S28]
MAF-49	39	38	2.7	0.78	[S29]
ZIF-4 [b]	52	48	2.2	0.83	[S30]
HOF-76[a]	66	37	2.05	-	[S22]
MUF-15[b]	105	93	1.96	1.53	[S31]
PCN-250	117	95	1.9	1.48	[S18]
PCN-245	73	54	1.8	0.8	[S32]
CPM-733	160	143	1.75	1.38	[S6]
ZIF-8[b]	45	34	1.7	0.56	[S33]
CPM-233	167	146	1.64	1.73	[S6]
JNU-2	92	81	1.6	0.55	[S34]
Ni(BDC)$(TED)_{0.5}$	112	76	1.6	1.01	[S35]
IRMOF-8	113	108	1.6	1.4	[S9]
Ni1a	148	136	1.5	1.3	[S36]
ZIF-7	41	40	1.5	0.94	[S37-38]
UTSA-33	62	61	1.4	0.43	[S21]
UTSA-35	55	49	1.4	0.37	[S39]
CPM-223-tppy	161	164	1.28	0.9	[S6]
TJT-100	81	75	1.2	0.73	[S20]

The calculations are based on the sorption data at 296 K (a) and 293 K (b).

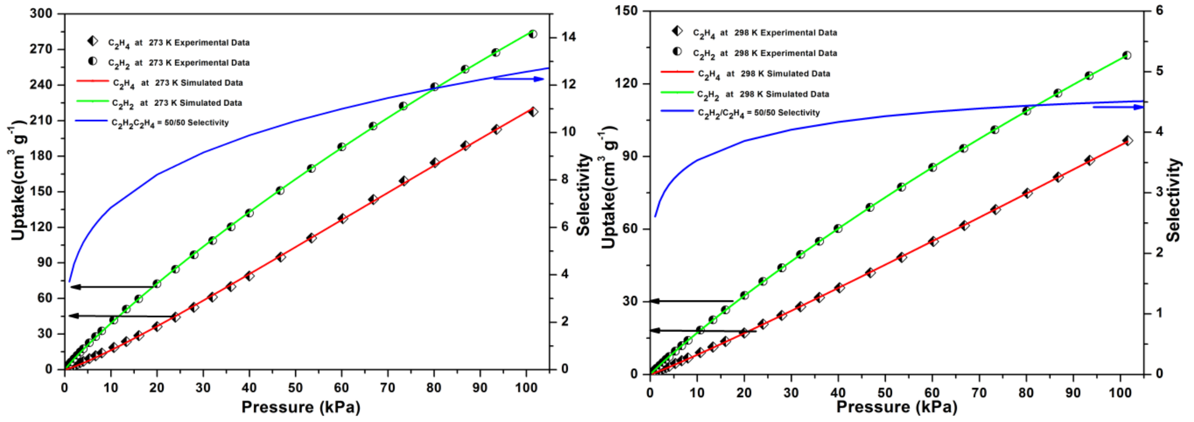
C_2H_4 at 273 K Experimental Data
C_2H_2 at 273 K Experimental Data
C_2H_4 at 273 K Simulated Data
C_2H_2 at 273 K Simulated Data
$C_2H_2C_2H_4$ = 50/50 Selectivity
C_2H_4 at 298 K Experimental Data
C_2H_2 at 298 K Experimental Data
C_2H_4 at 298 K Simulated Data
C_2H_2 at 298 K Simulated Data
C_2H_2/C_2H_4 = 50/50 Selectivity
Uptake(cm^3 g^{-1})
Selectivity
Pressure (kPa)

(a)

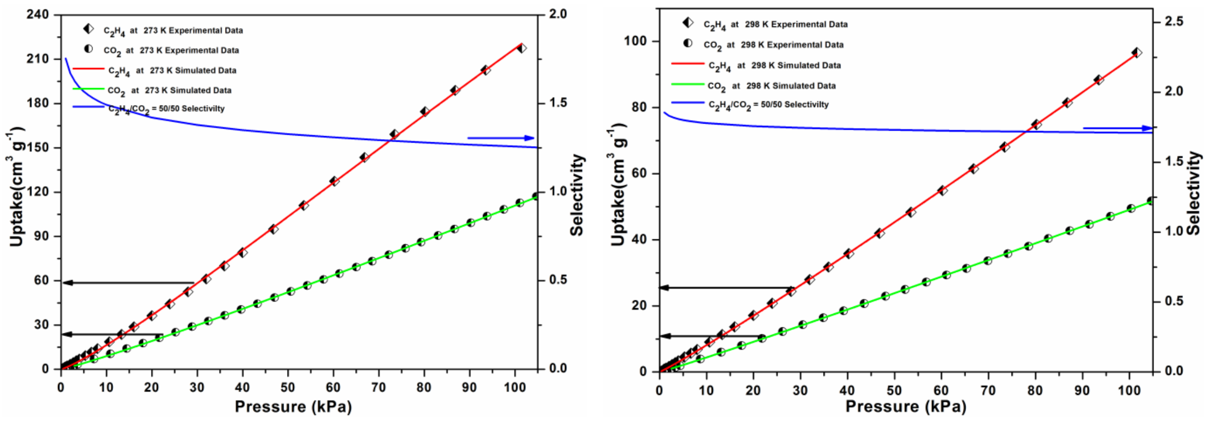
C_2H_4 at 273 K Experimental Data
CO_2 at 273 K Experimental Data
C_2H_4 at 273 K Simulated Data
CO_2 at 273 K Simulated Data
C_2H_4/CO_2 = 50/50 Selectivity
C_2H_4 at 298 K Experimental Data
CO_2 at 298 K Experimental Data
C_2H_4 at 298 K Simulated Data
CO_2 at 298 K Simulated Data
C_2H_4/CO_2 = 50/50 Selectivity
Uptake(cm^3 g^{-1})
Selectivity
Pressure (kPa)

(b)

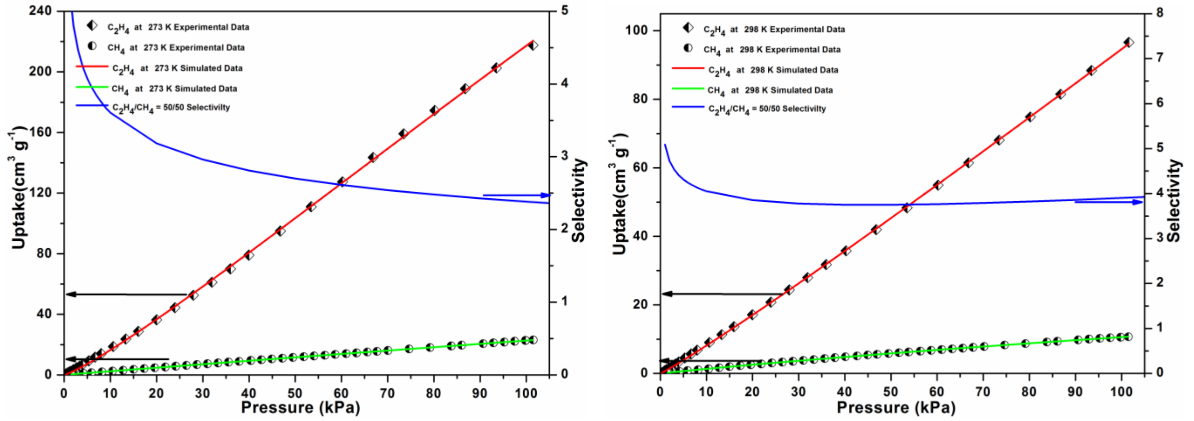
C_2H_4 at 273 K Experimental Data
CH_4 at 273 K Experimental Data
C_2H_4 at 273 K Simulated Data
CH_4 at 273 K Simulated Data
C_2H_4/CH_4 = 50/50 Selectivity
C_2H_4 at 298 K Experimental Data
CH_4 at 298 K Experimental Data
C_2H_4 at 298 K Simulated Data
CH_4 at 298 K Simulated Data
C_2H_4/CH_4 = 50/50 Selectivity
Uptake(cm^3 g^{-1})
Selectivity
Pressure (kPa)

(c)

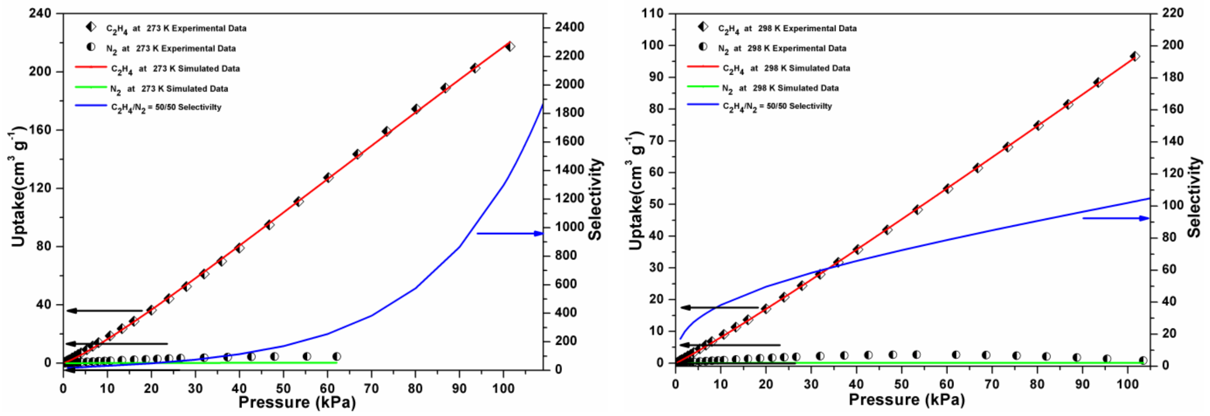

(d)

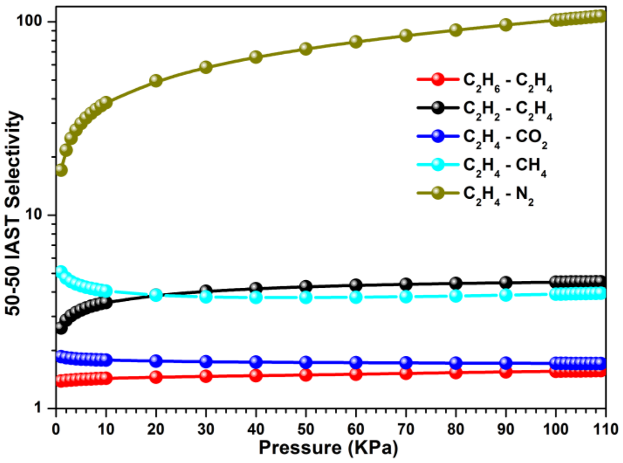

(e)

Figure S12. Comparison of experimental isotherms and simulated isotherms (Left Y axis), and mixture adsorption selectivity predicted by IAST (Right Y axis) of SNNU-40 for equimolar binary-mixture at 273 K and 298 K: (a) C_2H_2/C_2H_4, (b) C_2H_4/CO_2, (c) C_2H_4/CH_4, (d) C_2H_4/N_2 and (e) The summary of IAST-calculated selectivities for C_2H_6-C_2H_4, C_2H_2-C_2H_4, C_2H_4-CO_2, C_2H_4-CH_4 and C_2H_4-N_2 in the equimolar binary gas mixtures at 298 K.

Section S8: Isosteric heats of adsorption for SNNU-40

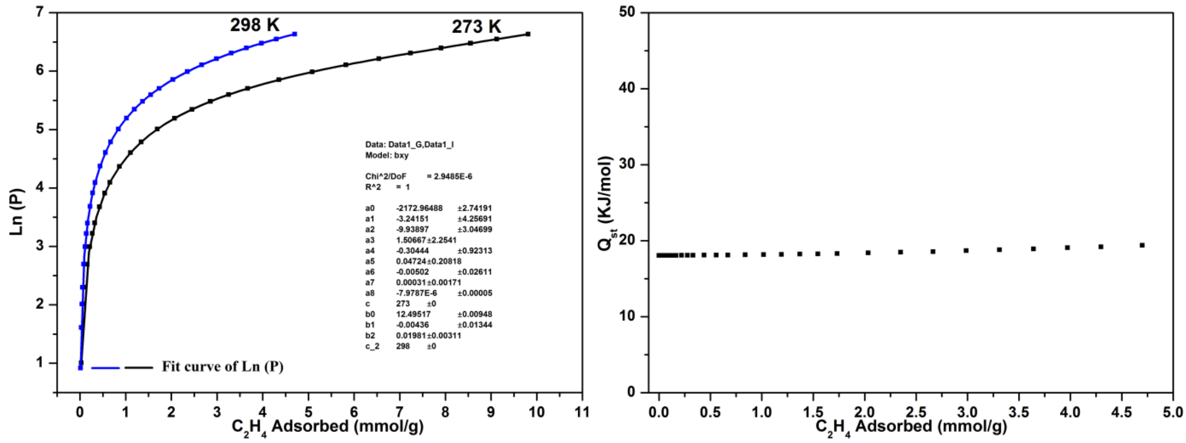

(a)

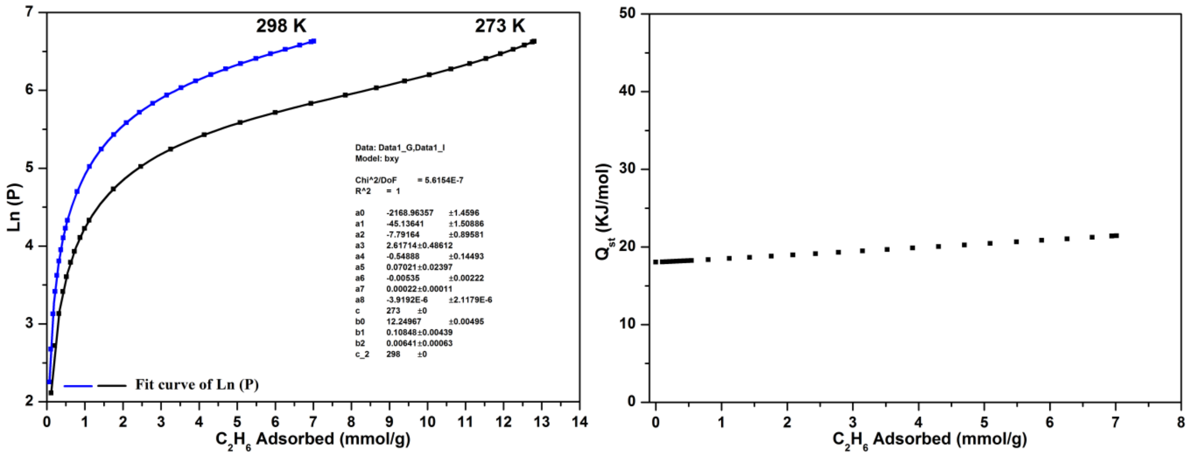

(b)

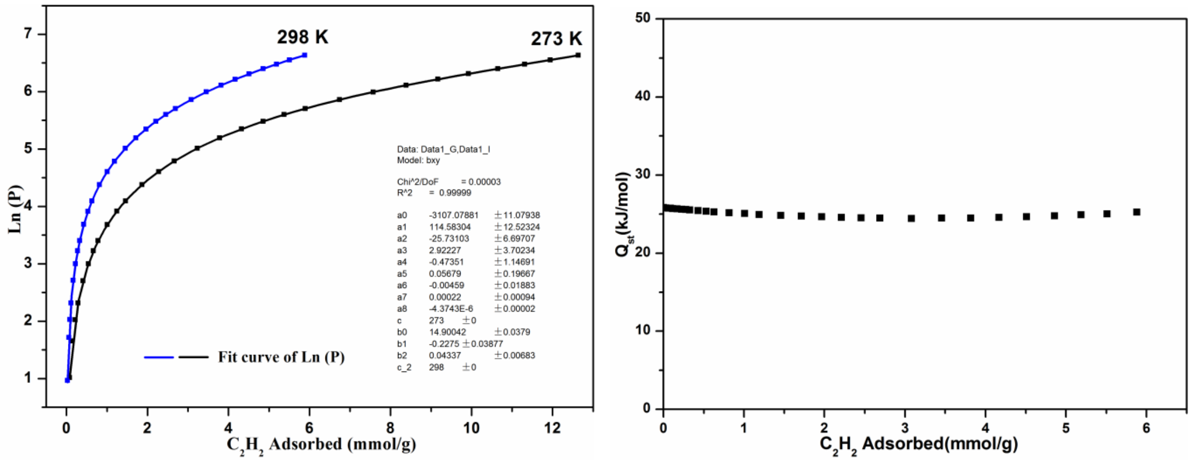

(c)

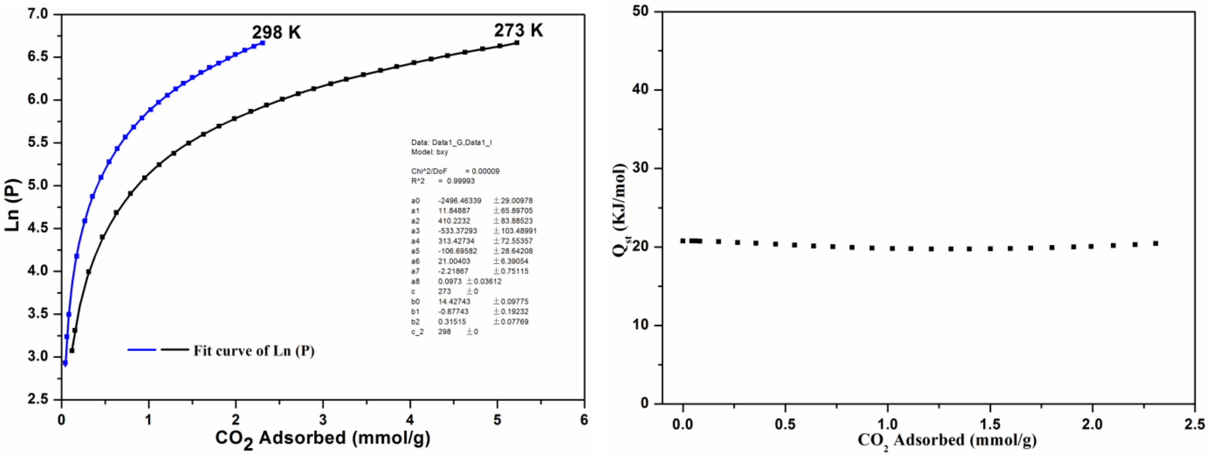

(d)

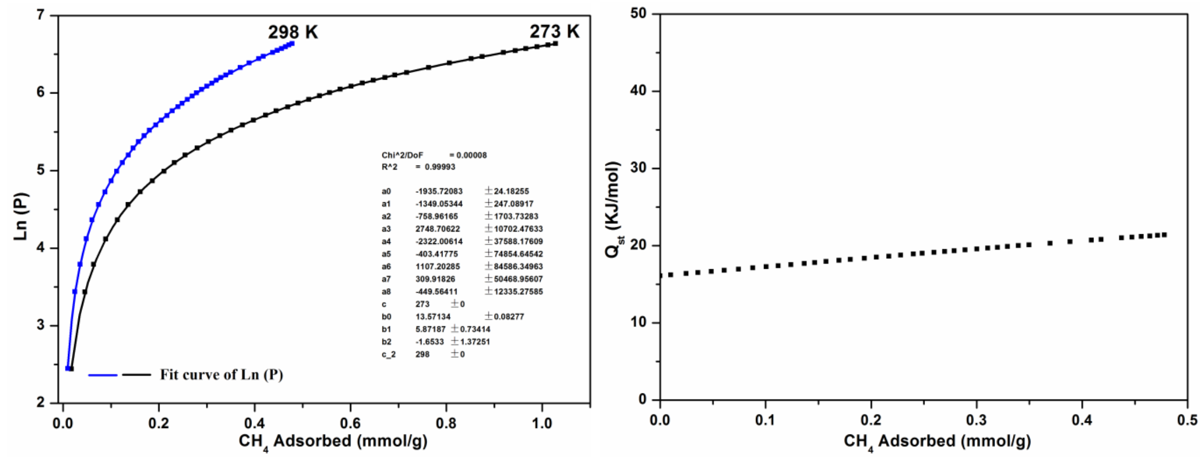

(e)

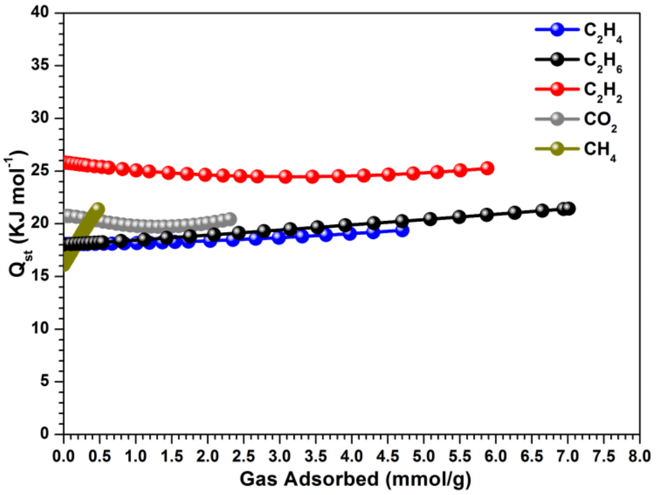

(f)

Figure S13. Fitted gas adsorption isotherms of SNNU-40 measured at 273 K and 298 K, and their corresponding isosteric heats of adsorption (Q_{st}).

Section S9: Column breakthrough experiments for SNNU-40

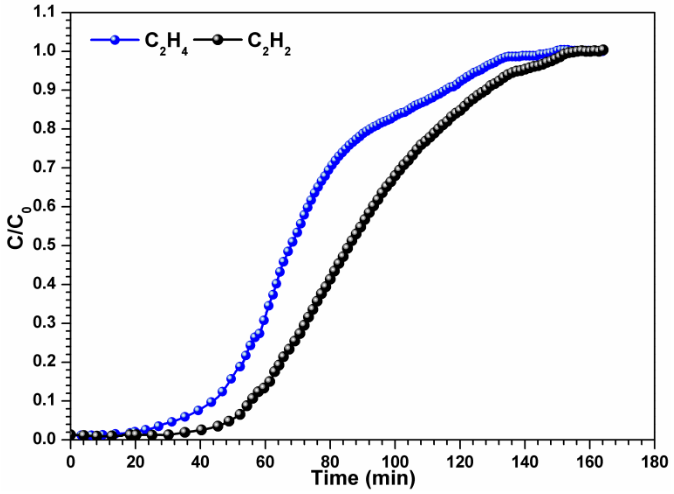

Figure S14. Column breakthrough experiments for C_2H_2/C_2H_4 (50/50, v/v, 2 mL/min) of SNNU-40 at 1 bar and 273 K.

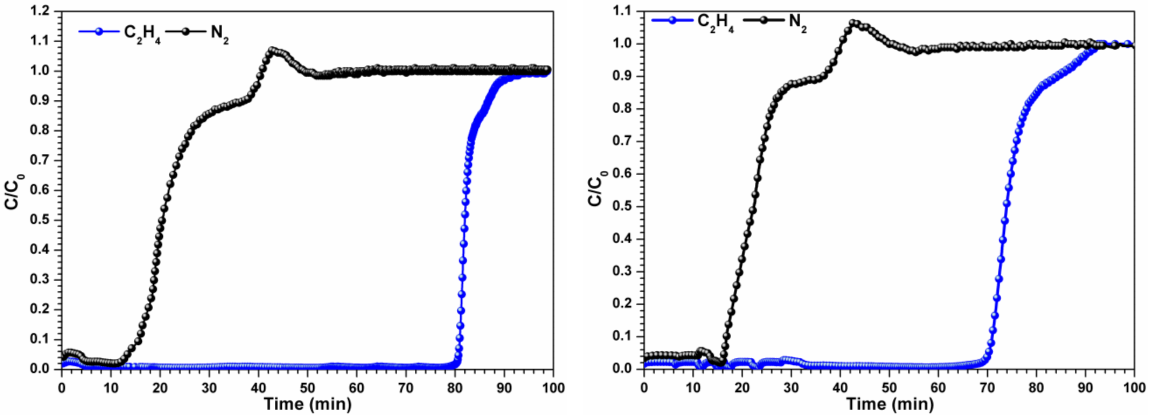

Figure S15. Column breakthrough experiments for C_2H_4/N_2 (50/50, v/v, 2mL/min) at 1 bar of SNNU-40: 273 K (left) and 298 K (right).

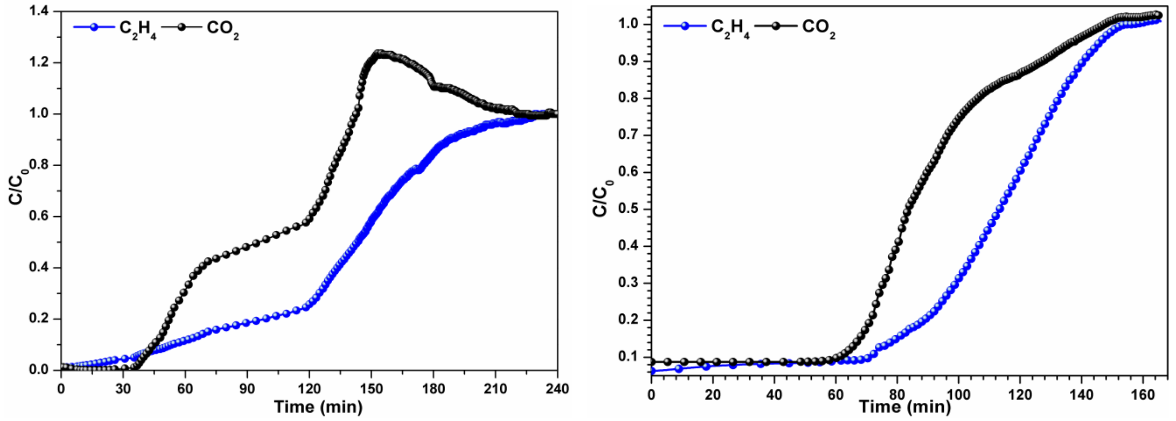

Figure S16. Column breakthrough experiments for C_2H_4/CO_2 (50/50, v/v, 2mL/min) at 1 bar of SNNU-40: 273 K (left) and 298 K (right).

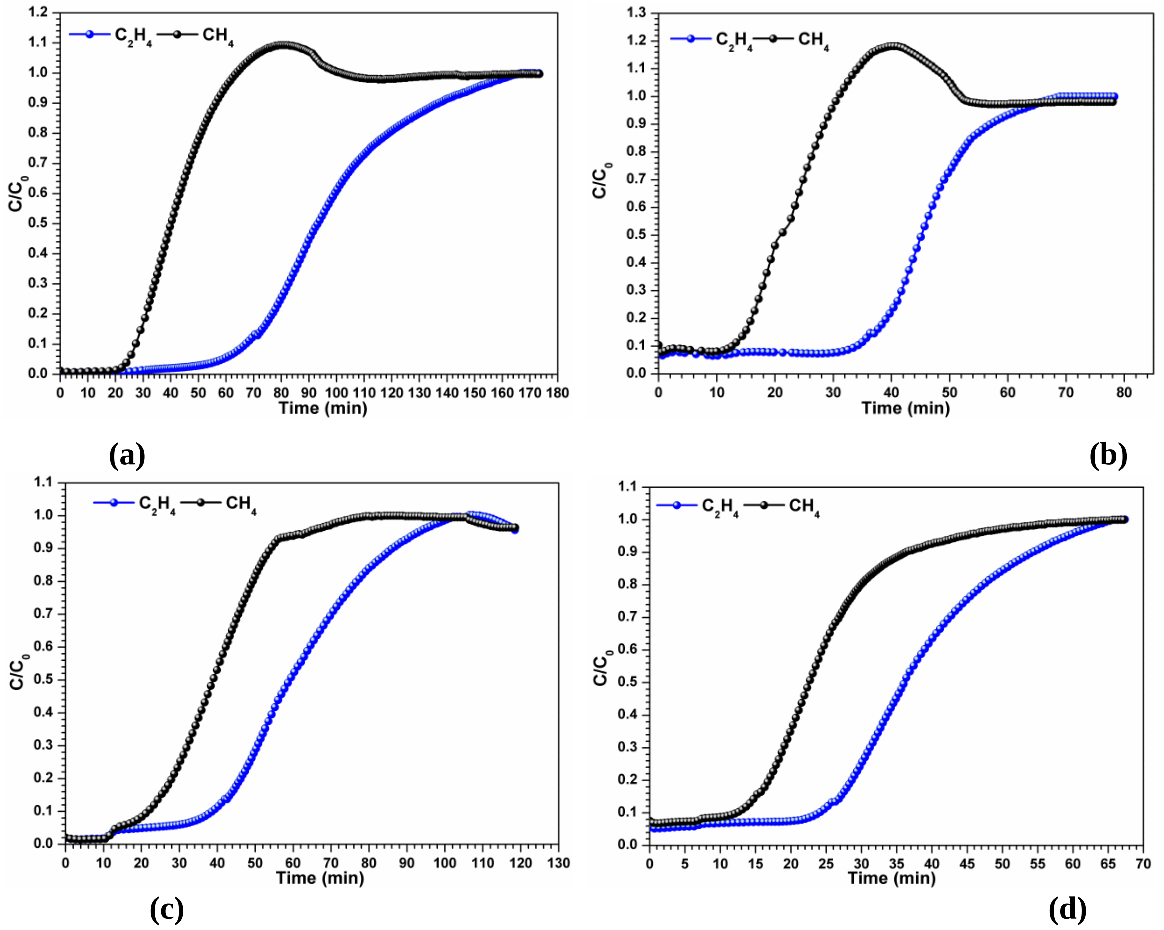

Figure S17. Column breakthrough experiments for C_2H_4/CH_4 (50/50, v/v, 2mL/min) at 1 bar and different temperature of SNNU-40: 263 K (a), 273 K (b), 283 K (c), and 298 K (d).

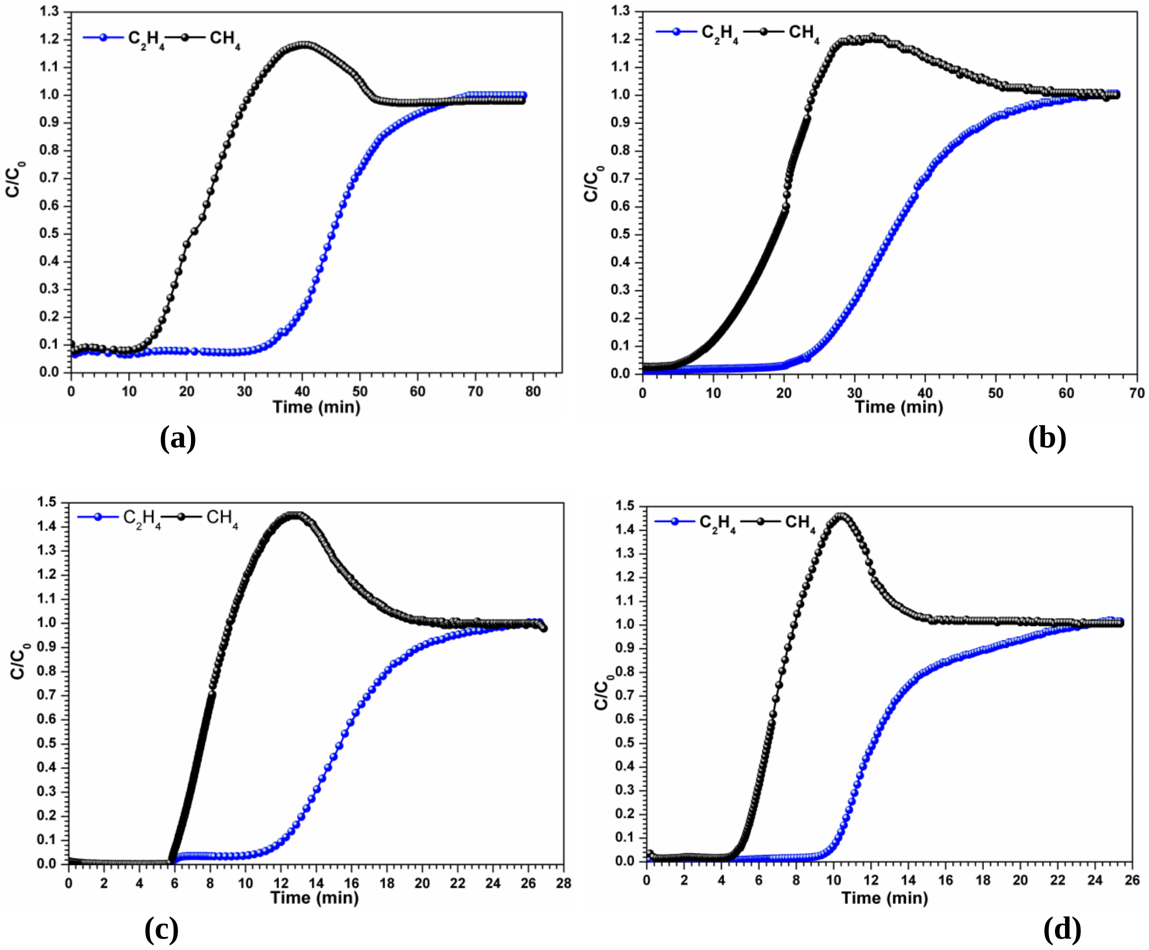

Figure S18. Column breakthrough experiments for C_2H_4/CH_4 (50/50,v/v) at 1 bar and 273K with different gas flow to SNNU-40: 2 mL/min (a), 4 mL/min (b), 6 mL/min (c), 8 mL/min (d).

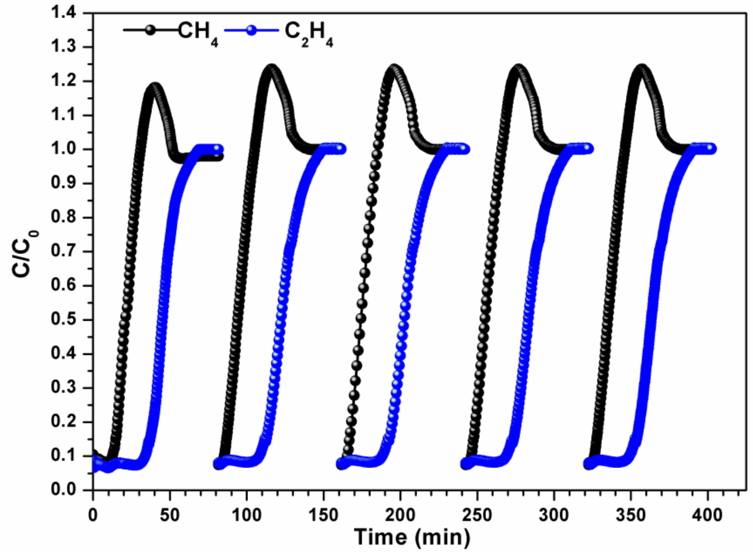

Figure S19. C_2H_4/CH_4 (50/50) separation repeat five cycles. Each separation process was carried out at 273 K and 1 bar with the regeneration performed by using a He flow (30 mL/min) for 30 min.

Section S11: References

[S1] B. Zhai, Z.-Y. Li, Z.-L. Wu, J.-Z. Cui, *Inorg. Chem. Commun.* **2016**, *71*, 23.

[S2] G. M. Sheldrick, *SHELXS 97:* Program for the Solution of Crystal Structure; *University of Göttingen: Göttingen, Germany*, **1997**.

[S3] G. M. Sheldrick, *SHELXS 97:* Program for the Crystal Structure Refinement; *University of Göttingen: Göttingen, Germany,* **1997**.

[S4] G. M. Sheldrick, SADABS: Siemens Area correction Absorption Program; *University of Göttingen: Göttingen, Germany*, **1994**.

[S5] R. Krishna, *RSC Adv.* **2017**, *7*, 35724.

[S6] H. Yang, Y. Wang, R. Krishna, X. Jia, Y. Wang, A. N. Hong, C. Dang, H. E. Castillo, X. Bu, P. Feng, *J. Am. Chem. Soc.* **2020**, *142*, 2222.

[S7] K. Liu, D. Ma, B. Li, Y. Li, K. Yao, Z. Zhang, Y. Han, Z. Shi, *J. Mater. Chem. A* **2014**, *2*, 15823.

[S8] Y. He, S. Xiang, B. Chen, *J. Am. Chem. Soc.* **2011**, *133*, 14570.

[S9] Y. He, R. Krishna, B. Chen, *Energy Environ. Sci.* **2012**, *5*, 9107.

[S10] D. Sun, S. Ma, J. M. Simmons, J.-R. Li, D. Yuan, H.-C. Zhou, *Chem. Commun.* **2010**, *46*, 1329.

[S11] K. Liu, D. Ma, B. Li, Y. Li, K. Yao, Z. Zhang, Y. Han, Z. Shi, *J. Mater. Chem. A* **2014**, *2*, 15823.

[S12] J.-W. Zhang, M.-C. Hu, S.-N. Li, Y.-C. Jiang, P. Qu, Q.-G. Zhai, *Chem. Commun.* **2018**, *54*, 2012.

[S13] X. Lin, J. Jia, X. Zhao, K. M. Thomas, A. J. Blake, G. S. Walker, N. R. Champness, P. Hubberstey, M. Schröder, *Angew. Chem., Int. Ed.* **2006**, *45*, 7358.

[S14] Z. Bao, S. Alnemrat, L. Yu, I. Vasiliev, Q. Ren, X. Lu, S. Deng, *Langmuir* **2011**, *27*, 13554.

[S15] X. Duan, Q. Zhang, J. Cai, Y. Yang, Y. Cui, Y. He, C. Wu, R. Krishna, B. Chen, G. Qian, *J. Mater. Chem. A* **2014**, *2*, 2628.

[S16] B. Chen, N. W. Ockwig, A. R. Millward, D. S. Contreras, O. M. Yaghi, *Angew. Chem., Int. Ed.* **2005**, *44*, 4745.

[S17] C.-X. Chen, S.-P. Zheng, Z.-W. Wei, C.-C. Cao, H.-P. Wang, D. Wang, J.-J. Jiang, D. Fenske, C.-Y. Su, *Chem. - Eur. J.* **2017**, *23*, 4060.

[S18] Y. Chen, Z. Qiao, H. Wu, D. Lv, R. Shi, Q. Xia, J. Zhou, Z. Li, *Chem. Eng. Sci.* **2018**, *175*, 110.

[S19] X. Wang, L. Li, Y. Wang, J.-R. Li, J. Li, *CrystEngComm* **2017**, *19*, 1729.

[S20] H. G. Hao, Y. F. Zhao, D. M. Chen, J. M. Yu, K. Tan, S. Ma, Y. Chabal, Z. M. Zhang, J. M. Dou, Z. H. Xiao, G. Day, H. C. Zhou, T. B. Lu, *Angew. Chem., Int. Ed.* **2018**, *57*, 16067.

[S21] Y. He, Z. Zhang, S. Xiang, F. R. Fronczek, R. Krishna, B. Chen, *Chem. - Eur. J.* **2012**, *18*, 613.

[S22] X. Zhang, L. Li, J.-X. Wang, H.-M. Wen, R. Krishna, H. Wu, W. Zhou, Z.-N. Chen, B. Li, G. Qian, B. Chen, *J. Am. Chem. Soc.* **2020**, *142*, 633.

[S23] L. Li, X. Wang, J. Liang, Y. Huang, H. Li, Z. Lin, R. Cao, *ACS. Appl. Mater. Interfaces* **2016**, *8*, 9777.

[S24] R.-B. Lin, H. Wu, L. Li, X.-L. Tang, Z. Li, J. Gao, H. Cui, W. Zhou, B. Chen, *J. Am. Chem. Soc.* **2018**, *140*, 12940.

[S25] X. Duan, H. Wang, Z. Ji, Y. Cui, Y. Yang, G. Qian, *Mater. Lett.* **2017**, *196*, 112.

[S26] X. Liu, W. Fan, M. Zhang, G. Li, H. Liu, D. Sun, L. Zhao, H. Zhu, W. Guo, *Mat. Chem. Front.* **2018**, *2*, 1146.

[S27] L. Li, R.-B. Lin, R. Krishna, H. Li, S. Xiang, H. Wu, J. Li, W. Zhou, B. Chen, *Science* **2018**, *362*, 443.

[S28] J. Pei, J.-X. Wang, K. Shao, Y. Yang, Y. Cui, H. Wu, W. Zhou, B. Li, G. Qian. *J. Mater. Chem. A* **2020**, *8*, 3613.

[S29] P.-Q. Liao, W.-X. Zhang, J.-P. Zhang, X.-M. Chen, *Nat. Commun.* **2015**, *6*, 8697.

[S30] M. Hartmann, U. Böhme, M. Hovestadt, C. Paula, *Langmuir* **2015**, *31*, 12382.

[S31] O. T. Qazvini, R. Babarao, Z.-L. Shi, Y.-B. Zhang, S. G. Telfer, *J. Am. Chem. Soc.* **2019**, *141*, 5014.

[S32] D. Lv, R. Shi, Y. Chen, Y. Wu, H. Wu, H. Xi, Q. Xia, Z. Li, *ACS. Appl. Mater. Interfaces* **2018**, *10*, 8366.

[S33] U. Böhme, B. Barth, C. Paula, A. Kuhnt, W. Schwieger, A. Mundstock, J. Caro, M. Hartmann, *Langmuir* **2013**, *29*, 8592.

[S34] H. Zeng, X.-J. Xie, M. Xie, Y.-L. Huang, D. Luo, T. Wang, Y. Zhao, W. Lu, D. Li, *J. Am. Chem. Soc.* **2019**, *141*, 20390.

[S35] W. Liang, F. Xu, X. Zhou, J. Xiao, Q. Xia, Y. Li, Z. Li, *Chem. Eng. Sci.* **2016**, *148*, 275.

[S36] H. Xiang, Y. Shao, A. Ameen, H. Chen, W. Yang, P. Gorgojo, F. R. Siperstein, X. Fan, Q. Pan, *Sep. Purif. Technol.* **2020**, *242*, 116819.

[S37] C. Gücüyener, J. van den Bergh, J. Gascon, F. Kapteijn, *J. Am. Chem. Soc.* **2010**, *132*, 17704.

[S38] D.-L. Chen, N. Wang, C. Xu, G. Tu, W. Zhu, R. Krishna, *Microporous Mesoporous Mater.* **2015**, *208*, 55.

[S39] Y. He, Z. Zhang, S. Xiang, F. R. Fronczek, R. Krishna, B. Chen, *Chem. Commun.* **2012**, *48*, 6493.
